# *Staphylococcus aureus* RnpA Inhibitors: Computational-Guided Design, Synthesis and Initial Biological Evaluation

**DOI:** 10.3390/antibiotics10040438

**Published:** 2021-04-14

**Authors:** Lorenzo Suigo, Michaelle Chojnacki, Carlo Zanotto, Victor Sebastián-Pérez, Carlo De Giuli Morghen, Andrea Casiraghi, Paul M. Dunman, Ermanno Valoti, Valentina Straniero

**Affiliations:** 1 Dipartimento di Scienze Farmaceutiche, Università degli Studi di Milano, Via Luigi Mangiagalli 25, 20133 Milano, Italy; lorenzo.suigo@unimi.it (L.S.); a.casiraghi.89@gmail.com (A.C.); ermanno.valoti@unimi.it (E.V.); 2 Department of Microbiology and Immunology, University of Rochester Medical Center, 601 Elmwood Ave., Rochester, NY 14642, USA; michaelle_chojnacki@urmc.Rochester.edu (M.C.); paul_dunman@urmc.rochester.edu (P.M.D.); 3 Dipartimento di Biotecnologie Mediche e Medicina Traslazionale, Università degli Studi di Milano, Via Vanvitelli 32, 20129 Milano, Italy; carlo.zanotto@unimi.it; 4 Exscientia, The Schrödinger Building, Oxford Science Park, Oxford OX4 4GE, UK; victorsebastianperez@gmail.com; 5 Department of Chemical—Pharmaceutical and Biomolecular Technologies, Catholic University “Our Lady of Good Counsel”, Rr. Dritan Hoxha, 1025 Tirana, Albania; carlo.degiulimorghen@unimi.it

**Keywords:** antibiotic resistance, RnpA inhibitors, MRSA, UAMS-1, RNA degradation machinery, antimicrobial activity, gram-positive bacteria

## Abstract

Antibiotic resistance is spreading worldwide and it has become one of the most important issues in modern medicine. In this context, the bacterial RNA degradation and processing machinery are essential processes for bacterial viability that may be exploited for antimicrobial therapy. In *Staphylococcus aureus*, RnpA has been hypothesized to be one of the main players in these mechanisms. *S. aureus* RnpA is able to modulate mRNA degradation and complex with a ribozyme (*rnpB*), facilitating ptRNA maturation. Corresponding small molecule screening campaigns have recently identified a few classes of RnpA inhibitors, and their structure activity relationship (SAR) has only been partially explored. Accordingly, in the present work, using computational modeling of *S. aureus* RnpA we identified putative crucial interactions of known RnpA inhibitors, and we used this information to design, synthesize, and biologically assess new potential RnpA inhibitors. The present results may be beneficial for the overall knowledge about RnpA inhibitors belonging to both RNPA2000-like thiosemicarbazides and JC-like piperidine carboxamides molecular classes. We evaluated the importance of the different key moieties, such as the dichlorophenyl and the piperidine of JC2, and the semithiocarbazide, the furan, and the *i*-propylphenyl ring of RNPA2000. Our efforts could provide a foundation for further computational-guided investigations.

## 1. Introduction

The well-documented consequences of antimicrobial resistance have been exacerbated by the limited pharmaceutical investment in new molecules [1], together with antibiotic over-prescription and misuse [2]. Indeed, antimicrobial resistance is currently surging in every country of the world, as reported by the World Health Organization (WHO) [3]. In this context, the resistance of several pathogens, such as *Klebsiella pneumoniae*, *Escherichia coli*, *Mycobacterium tuberculosis*, and *Plasmodium falciparum*, to both front line and antibiotics of last resort, are progressively leading to higher incidences of morbidity and mortality, as well as increased healthcare costs that are related to prolonged stays in hospitals [4]. Furthermore, because most of the commercially available antibiotics belong to the same structural classes, the development of resistance to a single agent can rapidly broaden to the entire structural class [5].

Among the pathogenic and highly virulent bacteria comprised in the ESKAPE classification (*Enterococcus faecium*, *Staphylococcus aureus*, *Klebsiella pneumoniae*, *Acinetobacter baumannii*, *Pseudomonas aeruginosa*, and *Enterobacter* spp.), methicillin-resistant *Staphylococcus aureus* is responsible for the majority of nosocomial infections worldwide [6,7,8], and it is associated with a 64% increase of death risk in comparison to the drug-sensitive strains [3]. In addition to methicillin, *S. aureus* has developed resistance to virtually all other front line antibiotics, including vancomycin [9], ceftaroline, linezolid, and daptomycin [8,10,11,12,13,14]. These alarming data, together with a limited antibiotic development pipeline [15], necessitate screening chemical space for agents that inhibit novel antimicrobial targets as a means to develop new chemical classes of antimicrobials.

Post-transcriptional regulation has recently been recognized as a crucial process in both eukaryotic [16] and prokaryotic cells for the control of gene expression [17,18,19]. In *S. aureus*, mRNA turnover is able to finely regulate the transient expression of several virulence factors, allowing for the bacterium to colonize the host [20]. In both *E. coli* and *S. aureus*, the degradosome is also required for the maturation of several stable RNA species (rRNA, tRNA) [17,21]. For these reasons, very recently, the enzymes catalyzing bacterial RNA processing and degradation have emerged as potential targets for the development of novel antimicrobials [20].

**Figure 1:**
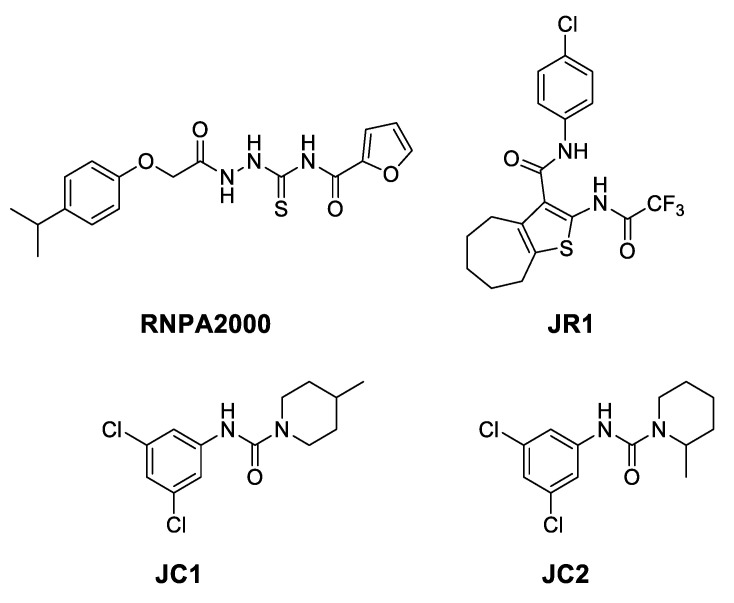
Structure of **RNPA2000**, **JC1**, **JC2**, and **JR1**, known RnpA inhibitors.

While in *E. coli*, the main player of the degradosome is the essential endoribonuclease RNase E [17,20], *S. aureus* does not contain an RNase E ortholog, rather the 13 KDa protein RnpA seems to contribute to cellular mRNA degradation as well as a second RNA-metabolic process. Indeed, RnpA has been shown to catalyze the digestion of mRNA and it can interact with the ribozyme *rnpB*, thus forming the RNase P riboprotein complex, which is responsible for the removal of the 5′ leader sequences from precursor tRNA (ptRNA), thus promoting tRNA maturation [20]. As such, the protein may be a novel antimicrobial target that is required for two essential processes, mRNA degradation and ptRNA processing, and allows for the identification of agents that inhibit either one or both processes. Eidem and co-workers developed and fully studied one of the first and most promising RnpA inhibitors, **RNPA2000** (Figure 1), which has been shown to inhibit RnpA mediated mRNA turnover and ptRNA maturation [22]. 

Starting from **RNPA2000**, Lounsbury and co-workers developed a series of analogues to overcome the structural weaknesses of this molecule, starting from the replacement of the 2-furanyl moiety [23]. In parallel, Colquhoun and colleagues discovered two additional structural classes of potential RnpA inhibitors, the piperidine carboxamides and phenylcarbamoyl cyclic thiophenes. Several compounds of these classes, such as **JC2** and **JR1** (Figure 1), demonstrated the ability to interfere with both RnpA-mediated mRNA degradation and ptRNA processing, with very low IC_50_ values and low mammalian cell cytotoxicity.

JC2 was also evaluated in vivo using a *Galleria melonella* model of *S. aureus* infection, and it proved to be able to reduce the mortality rate at 16 h to 30%, which was comparable to the antibiotic vancomycin [24]. Yet, our understanding of the structure activity relationship (SAR) from previously described RnpA inhibitors remains at its infancy.

**Figure 2:**
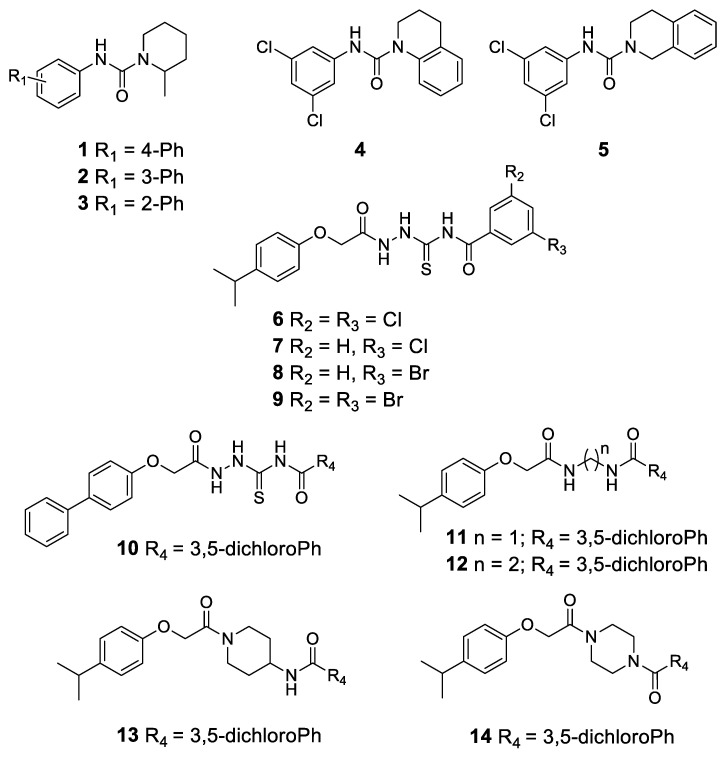
Structures of compounds **1**–**14**, object of the present work.

Therefore, expanding the SAR of the RnpA inhibitors is the main aim of the present work. Guided by a novel computational approach to predict the protein/compound binding sites, we designed and synthesized 14 **RNPA2000** or **JC1**/**JC2** derivatives (Figure 2, **1**–**14**) to probe key features of each chemical scaffold.

Compounds **1**–**5** aimed to assess the importance of each single moiety of **JC1/JC2**, where compounds **1**–**3** bear, in lieu of the 3,5-dichlorophenyl scaffold, a biphenylic one. As a result, we could evaluate the importance of the phenylic ring and, at the same time, we evaluated whether a bulkier, more lipophilic, and electron rich substituent could be well tolerated by the binding site. On the other hand, derivatives **4** and **5** were designed to investigate the opposite end of the molecule, by replacing the piperidine ring with a tetrahydroquinoline (**4**) or a tetrahydroisoquinoline (**5**).

Following the idea of Lounsbury and co-workers, to assess the relevance of the 2-furanyl moiety of **RNPA2000** [23], we also decided to evaluate some alternatives to the furanyl ring. Compounds **6**–**9** present phenyl rings, which are substituted with halogens: 3,5-dichloro (**6**), 3-chloro (**7**), 3-bromo (**8**), and 3,5-dibromo (**9**). We decided to mimic the same phenylic moiety of **JC1**/**JC2**, to appraise whether its specific electronic and steric combinations are required for a strong inhibition of RnpA. Moreover, the productivity of the substitution of heterocyclic moieties with halogen-phenyl rings has been widely demonstrated [25]. The derivative **10** presents the 4-biphenyl-moiety in lieu of the 4-*i*-propylphenyl one, as in compounds **1**–**3**. Finally, compounds **11**–**14** are characterized by differences in the linker between the two aromatic moieties. They were designed to evaluate the importance of the semi thiocarbazide moiety of **RNPA2000** in terms of electronic density, linearity, polarity, and conformational freedom.

## 2. Results and Discussion

### 2.1. Computational Studies and Design

Using *in-silico* predictions, we aimed to estimate which cavity of the protein could be responsible for the binding of **JC-like compounds**, **RNPA2000**, the set of derivatives synthesized in this work and to decipher the main key interactions of these compounds in the binding site.

**Figure 3:**
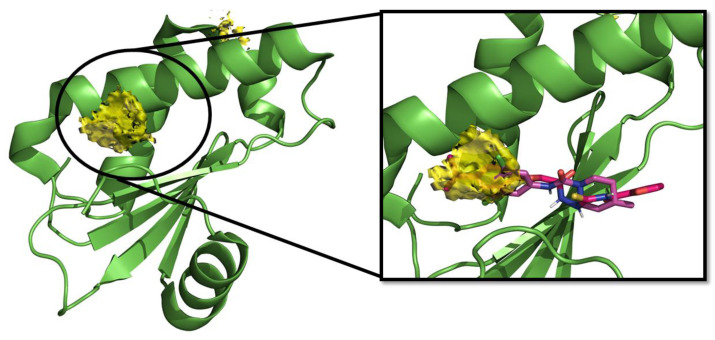
Hotspot maps calculated for the protein. Overlay between the hotspot maps and the predicted binding mode for **RNPA2000** and **JC1** in the hypothesized binding site.

With this objective and using *S. aureus* RnpA protein, a detailed Hotspots Maps calculation was performed. These results revealed that RnpA displays low druggability when compared with other proteins, and only one low hotspot scoring area was identified as the main ligandable binding site. Both **JC1** and **RNPA2000** were docked in this hypothetical binding pocket in order to validate our hypothesis (Figure 3).

First, and given the unknown mechanism of action of RnpA inhibitors in RNA degradation and ptRNA maturation, a blind docking study was carried out. For this study, we considered the RnpA structure, and two reference compounds that were proposed in the literature as RnpA inhibitors (JC1 and RNPA2000) described above to assess their predicted binding cavity. The docking studies suggest that the reference molecules might be able to bind RnpA in the main area that was identified previously in the hotspots maps. The top part of this region involves the sequence of highest phylogenetic conservation in the RnpA proteins being named the RNR box motif and represented by residues 59–67. This positive-charged motif, containing three arginines and two lysines, resides at the top half of α_2_ being solvent exposed, being poised to form the required interactions with the catalytic RNA ribozyme (P-RNA) component of the complex [26].

Specifically, there are several key subareas in the solvent-exposed pocket that might drive the ligand-protein interaction:

- in the subregion where the main hydrophobic hotspot was identified, there are several aromatic and hydrophobic residues, including Tyr7, Phe43, Leu45, and Phe70, where the first two are generating a cleft. This might help in anchoring aromatic systems, establishing π or CH–π interactions (such as the *i*-propylphenyl group that is present in RnpA2000);
- the central part contains a solvent-exposed area, which might allow the binding of linear linkers by establishing hydrogen bonds interactions via the backbone of Ile9, Leu45, Ile47 or the guanidinic moiety of Arg67. H-bonds donor-acceptor that are predicted to interact with the ureidic portion of JC1 and with the semi-thiocarbazide moiety of RNPA2000; and,
- finally, at the other end, Phe15 and Lys63 are suitable for π interaction or π-cation interactions with different aromatic moieties, such as the one present in RNA2000.

**Figure 4:**
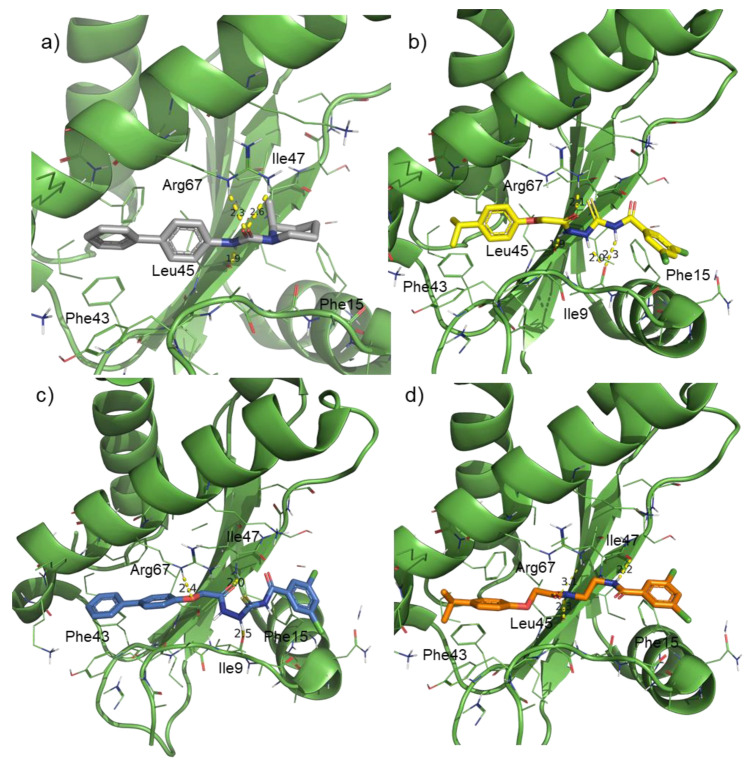
Predicted binding poses, based on docking studies, for different compounds designed in this work: (**a**) Compound **1** (light grey) docked in the putative binding site containing a biphenyl scaffold being the amide able to interact via H-bond with Leu45 and Arg67. (**b**) Compound **6** (yellow) where the carbonyl interacts with Arg67 and thiosemicarbazide makes H-bond with Ile9 and Leu45. (**c**) Compound **10** (blue) making H-bond interaction with Ile9 and Ile47, and Arg67. (**d**) Compound **11** (orange) making H-bond interaction with Leu45, Ile47, and Arg67 with the double amide moiety.

According to the docking results, compound **1** is predicted to bind the same cavity achieving interactions that are close to the reference compounds mentioned above (Figure 4a). The biphenyl motif engages with the aromatic residues on the cleft, while the amide makes H-bond with Leu45, and Arg67, as shown in Figure 4a. On the other hand, **2** and **3** with a different substitution pattern in the biphenyl system and with the vector growing at the 2 and 3 positions towards the solvent exposed area seem to be less attractive to fit in the cleft region as compared to **1**.

Compounds **4** and **5** were designed to potentially achieve an extra-interaction with the Phe15 or Lys63 at the binding site, by the substitution of the piperidine ring of **JC1** with a tetrahydroquinoline or a tetrahydroisoquinoline, respectively. Compounds **6**–**9** showed a very good interaction profile and fit in the pocket, with the *i*-propylphenyl and the semi-thiocarbazide showing a very similar shape to RNPA2000 and an optimal linearity given by the linker. At the other end, the replacement of the furanyl moiety by different halogen-phenyl systems are tolerated, as depicted in Figure 4b with compound **6**. While considering derivative **10**, the biphenylic portion was introduced to replace the characteristic *i*-propylic substituent present in other RNPA2000-like structures and evaluate its effects. Compound **10** keeps the H-bond interactions between the linker and Ile9, Ile47 and Arg67 and the biphenyl achieves a perfect fit in the cleft (Figure 4c).

Finally, compounds **11**–**14** were designed to evaluate the importance of the semi-thiocarbazide moiety. Even removing the semi-thiocarbazide and replacing it with a di-amidic structure, these compounds maintain some of the key interactions with RnpA, as shown in Figure 4d. Moreover, this series of compounds should be more metabolically stable than RNPA2000-like derivatives, which might contain a liable nitrogen-nitrogen bond.

From a computational point of view, the hotspot maps analysis, together with the docking studies, suggested the binding cavity for this set of compounds proposing their putative binding mode and their main interactions with RnpA. In addition, several structural modifications were proposed to test different hypothesis and evaluate the effect of these modifications on both the phenotypic assay and the capability of these compounds to inhibit in-vitro RnpA-mediated ptRNA maturation. These results will need further structural confirmation in future studies, given the lack of structural information for inhibitors of this route.

### 2.2. Chemistry

**Scheme 1:**
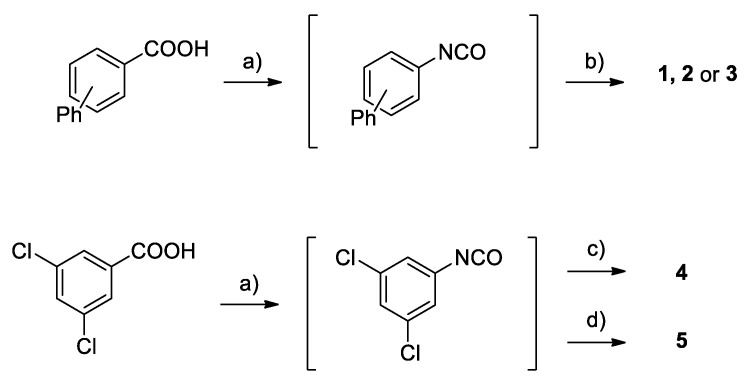
*Reagents and solvents:* (**a**) Diphenyl phosphoryl azide (DPPA), Triethylamine (TEA), toluene, 95 °C, 40 min; (**b**) 2-methylpiperidine, toluene, 95 °C; (**c**) 1,2,3,4-tetrahydroquinoline, toluene, 95 °C; and, (**d**) 1,2,3,4-tetrahydroisoquinoline, toluene, 95 °C.

Scheme 1 shows the synthesis of compounds **1** to **5**. The synthetic route is the same for each compound. The proper carboxylic acid was converted into the corresponding isocyanate by treatment with diphenyl phosphoryl azide and subsequent Curtius rearrangement after heating, and soon reacted with the appropriate amine, yielding the desired ureidic derivative.

**Scheme 2:**
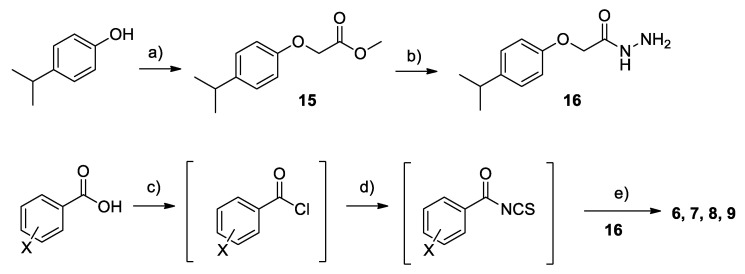
*Reagents and solvents:* (**a**) Methyl 2-chloroacetate, K_2_CO_3_, DMF, 50 °C, 1.5 h; (**b**) Hydrazine hydrate, Methanol, Reflux, 16 h; (**c**) SOCl_2_, Reflux, 1 h; (**d**) Potassium thiocyanate, Acetonitrile, room temperature (RT), 1 h; and, (**e**) Acetonitrile, RT, 30 min.

For the obtainment of the final compounds **6** to **9** (Scheme 2), we followed the synthetic route that was described by Lounsbury and colleagues [23], in which the key intermediate 2-(*i*-propylphenoxy)acetohydrazide is treated with the proper acyl isothiocyanate to obtain the thiosemicarbazide group, peculiar for this four compounds. To do so, we achieved the key intermediate **16** starting from the commercially available 4-*i*-propylphenol, which was alkylated by reaction with methyl 2-chloroacetate in the presence of K_2_CO_3_ as base and subsequently transformed into the corresponding hydrazide by treatment with hydrazine hydrate in methanol.

**Scheme 3:**
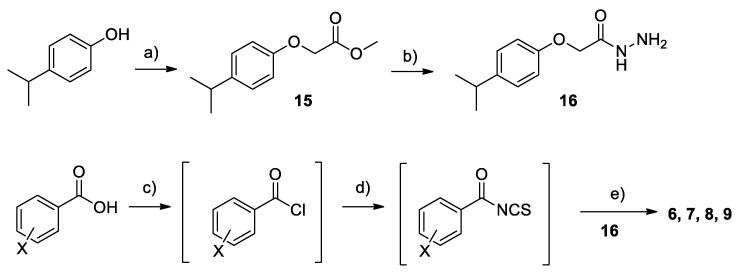
*Reagents and solvents:* (**a**) Methyl 2-chloroacetate, K_2_CO_3_, DMF, 50 °C, 1.5 h; (**b**) Hydrazine hydrate, methanol, reflux, 16 h; (**c**) SOCl_2_, reflux, 1 h; (**d**) Potassium thiocyanate, acetonitrile, RT, 1 h; and, (**e**) Acetonitrile, RT, 30 min.

The synthetic scheme that was performed for the synthesis of compound **10** (Scheme 3) follows the one just described for compounds **6** to **9**. In this case, the 4-phenylphenol was converted into the 2-(4-biphenyloxy)acetohydrazide, tracing the same strategy described before, and treated with the 3,5-dichlorobenzoyl isothiocyanate, yielding the desired compound **10**.

**Scheme 4:**
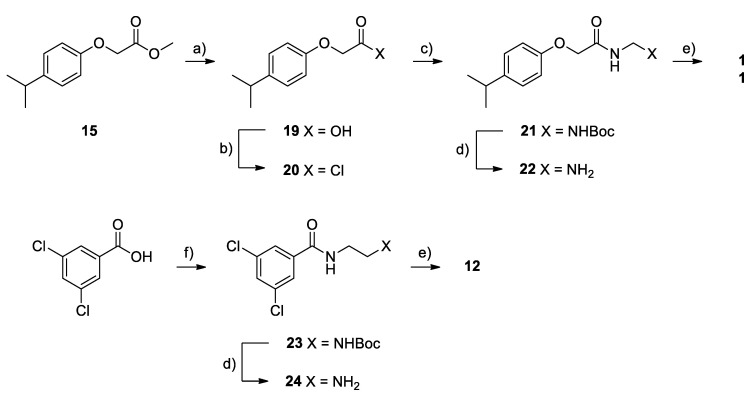
*Reagents and solvents:* (**a**) 2.5 N aqueous NaOH, MeOH, RT, 1.5 h; (**b**) SOCl_2_, reflux, 1 h; (**c**) *N*-Boc methylenediamine, TEA, DCM, RT, 3 h; (**d**) 10% aqueous HCl, MeOH, reflux, 0.5 h; (**e**) 3,5-dichlorobenzoyl chloride, TEA, DCM, RT, 1.5 h; (**f**) *N*-Boc ethylenediamine, TEA, DCM, RT 3 h, ad (**e**) Intermediate **20**, TEA, DCM, RT, 1 h.

The synthesis of compounds **11**–**12** (Scheme 4), characterized by two amidic groups linked at the nitrogen atoms by methylenic or ethylenic linker (compounds **11** and **12**), is based on the same strategy. Firstly, the two moieties were reacted with the appropriate monoprotected amine, generating the first amidic group. Thus, the intermediate was deprotected and linked to the second moiety to achieve the second amidic group. In particular, for compound **11**, the process started from intermediate **15**, which was hydrolyzed and treated with the *N*-Boc-methylendiamine, being adequately prepared, as described by DeBons and Loudon [27]. Therefore, this intermediate was deprotected and converted into the desired compound **11**. For compound **12**, conversely, the synthesis started from the 3,5-dichlorobenzoic acid, which undergoes the same reactions as before, using the appropriate amine.

**Scheme 5:**
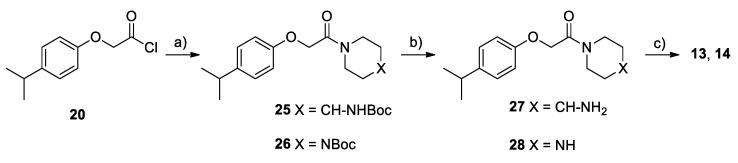
*Reagents and solvents:* (**a**) 4-*N*-Boc-aminopiperidine or *N*-Boc-piperazine, TEA, DCM, RT, 3 h; (**b**) TFA, DCM, RT, 18 h; (**c**) 3,5-dichlorobenzoyl chloride, TEA, DCM, RT, 2 h.

Lastly, the obtainment of compounds **13**–**14** (Scheme 5) was possible by following the same strategy previously described, since also these two derivatives are characterized by a diamidic structure. In this case, the intermediate **20** was treated with the corresponding amine, 4-*N*-Boc-aminopiperidine and *N*-Boc-piperazine, to achieve the first amidic group (**25**–**26**). Therefore, these intermediates were deprotected (**27**–**28**) and treated with 3,5-dichlorobenzoyl chloride yielding the final derivatives.

### 2.3. Biological Evaluation

#### 2.3.1. Antimicrobial Activity

The antimicrobial activities of compounds **1**–**14** (Table 1) were evaluated on two *S. aureus* strains: a methicillin-sensitive *S. aureus* (MSSA, ATCC 29213) and a methicillin-resistant *S. aureus* (MRSA, ATCC 43300). The inhibitory ability of compounds **1**–**14** was evaluated by determining the minimum inhibitory concentration (MIC).

Some of the tested compounds displayed very promising antimicrobial activities, with reasonable MICs (**6**, **7**, **8**, **9**, and **10**), as shown in Table 1. Next, we assessed whether these antimicrobial activities correlated with the inhibition of RnpA activities.

#### 2.3.2. In-Vitro Assays

**Figure 5:**
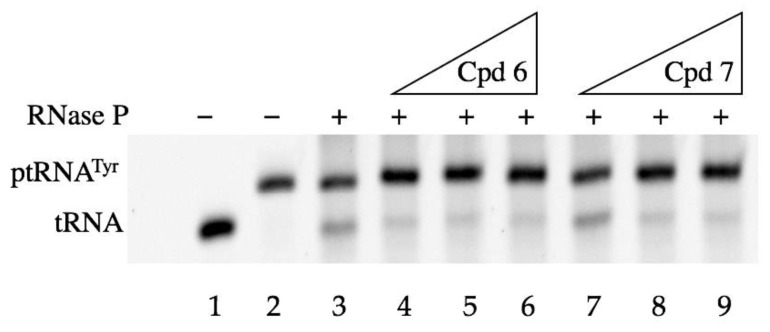
In-vitro ptRNA maturation assay for Compounds **6** and **7**. 0, 62.5, 250, or 500 µM compound was incubated with 10 pmol RNase P and ptRNA^Tyr^ for 30 min at 37 °C and conversion of ptRNA to tRNA was evaluated by urea-PAGE; migration of tRNA^Tyr^ and ptRNA^Tyr^ are shown.

Firstly, we evaluated the capability of these compounds to inhibit in-vitro RnpA-mediated ptRNA maturation. To do so, Rnase P (RnpA + *rnpB*) was reconstituted and combined with the putative inhibitor or DMSO (as a negative control) and ptRNA^Tyr^ for 30 min, and the relative quantities of ptRNA^Tyr^ and mature tRNA were evaluated by Urea-PAGE. Figure 5 depicts representative data of the performance of compounds **6** and **7**. The presence of RNase P alone (Figure 5, lane 3) catalyzes the conversion of ptRNA into mature tRNA. At the same time, increasing amounts of compounds **6** and **7** partially perturb the activity of RNase P, resulting in the accumulation of ptRNA (Figure 5, lanes 4 to 9). All of the compounds were tested at least twice and Table 2 records their IC_50_ values.

**Figure 6:**
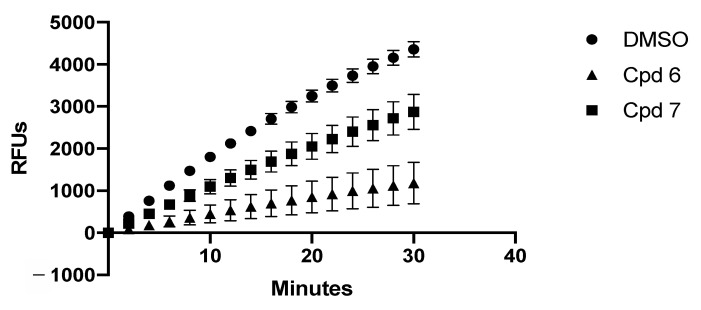
In-vitro mRNA degradation assay—Compounds **6** and **7**. Representative analogue inhibition of in-vitro RnpA-mediated RNA degradation. DMSO or 62.5 µM of the compound was incubated with RnpA in the presence of the FRET RNA substrate for 30 min at 37 °C. Fluorescence measurements (RFUs) were collected every 2 min at excitation 490 nm, emission 520 nm.

In parallel, the mRNA degradation inhibition profile of each compound was also evaluated. The time course experiments were performed by incubating 62.5 µM of the compound (or DMSO) with RnpA in the presence of a fluorescently labeled RNA substrate for 30 min. RnpA efficiently degraded RNA in the presence of DMSO (circles), but it was inhibited to 40–60% in the presence of the compounds **6** and **7**, as shown in Figure 6 (triangles and squares, respectively). Subsequent dose response assays were carried out using increasing concentrations of each analogue to determine the IC_50_ value of each compound (Table 2).

Table 2 summarizes the IC_50_ values for each compound, which were calculated for both mRNA degradation inhibition and ptRNA maturation inhibition. The results revealed that the substitution of the 3,5-dichlorophenyl moiety of **JC1**/**JC2** with a biphenylic one is always detrimental for the inhibition of the RnpA-mediated processes and, consequently, for the antimicrobial activity, although the 4-biphenilic derivative **1** presents a better overall profile when compared to the 3-biphenilic and the 2-biphenilic derivatives **2** and **3**. These data match with the in-silico prediction. Conversely, the tetrahydroquinolinic compound **4** shows stronger RnpA inhibition properties as compared to the tetrahydroisoquilinic derivative **5**. Despite that these results seem to be in contrast with the predicted docking data, the different activity might be due to other factors rather that the inability to correctly interact with RnpA. The IC_50_ values of compounds **6**, **7**, **8**, and, partially, **9** confirm the productivity of the substitution of the 2-furanylmoiety with halogen-phenyl rings. In particular, the dichlorophenyl derivative **6** present comparative inhibition profiles for mRNA degradation and ptRNA processing, while derivatives **7** (*m*-chloro) and **8** (*m*-bromo) show, respectively, better ptRNA processing inhibition and mRNA degradation inhibition capability. Compounds **11**–**14** present no antimicrobial activity, which suggests the importance of the semi-thiocarbazide moiety.

#### 2.3.3. Cellular Assays

**Figure 7:**
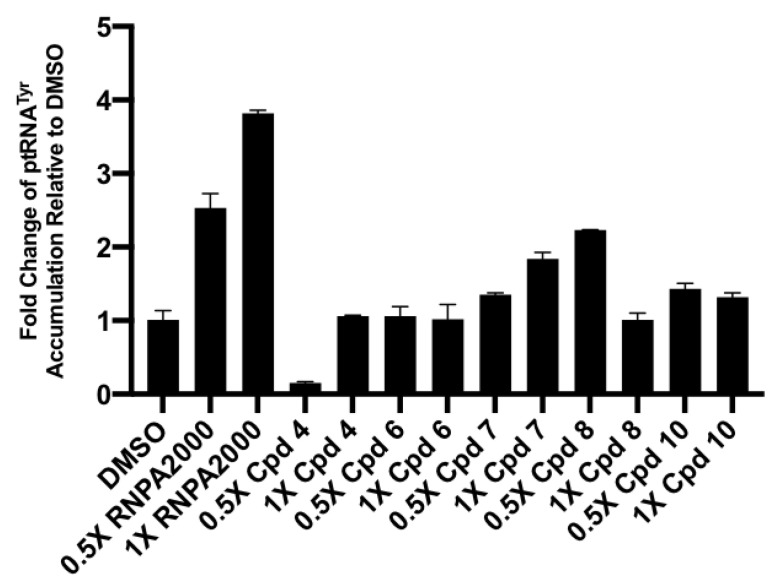
ptRNA accumulation cellular assay. Quantitative RT-PCR measures of ptRNA^Tyr^ accumulation in *S. aureus* UAMS-1 cells treated with DMSO, analogues or RNPA2000 (positive control). Cells were treated with 0.5× or 1× MIC for 1 h. Each compound was evaluated twice, and the values were averaged.

*S. aureus* were treated with 0.5× or 1× MIC of each compound for 1 h and qRT-PCR was used to quantify the accumulation of ptRNA^tyr^ in order to evaluate the capability of each compound to affect RnpA-mediated ptRNA processing in the more complex cellular system, as previously described [22,24]. Cells that were treated with DMSO did not accumulate ptRNA^Tyr^, whereas RNPA2000 (positive control) resulted in a dose-dependent accumulation of 2.5-fold and 3.8-fold at 0.5× and 1× MIC, respectively, as shown in Figure 7. Compound **7** confirmed its ability to inhibit RnpA-mediated ptRNA^Tyr^ processing and also demonstrated a dose-dependent effect on ptRNA^Tyr^ accumulation. Treatment with compound **8**, which had inhibitory activity in vitro, resulting in a 2.23-fold increase in ptRNA^Tyr^ accumulation at 0.5× MIC, but did not inhibit ptRNA^Tyr^ processing after treatment with 1× MIC, possibly due to the compound precipitation at this concentration. Compound **10**, which had a good in-vitro inhibition of ptRNA^Tyr^ processing, resulted in 1.4-fold and 1.3-fold increases in ptRNA^Tyr^ accumulation at 0.5× and 1× of the MIC, respectively. Treatment with compounds **4** and **6** did not result in the accumulation of ptRNA^Tyr^, despite the antistaphylococcal and inhibitory activity in vitro.

**Figure 8:**
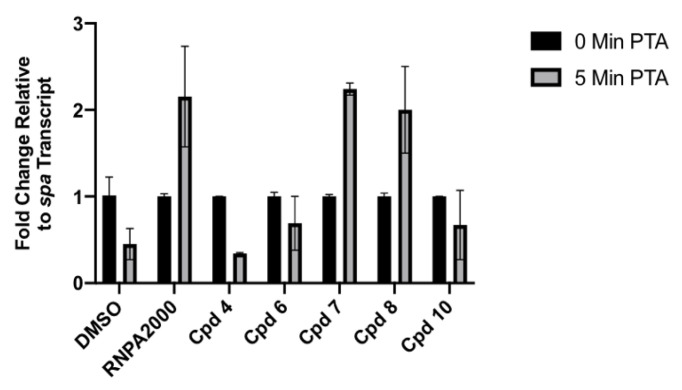
RT-PCR of *spa* transcript turnover. *S. aureus* UAMS-1 cells were treated with 0.5× MIC of each compound for 30 min prior to transcriptional arrest (PTA). Each compound was evaluated twice, and values were averaged.

As a more direct measure of the effect of each analogue, on cellular mRNA degradation, RnpA-mediated turnover of *spa* RNA, a natural substrate of RnpA, was evaluated. *S. aureus* UAMS-1 was treated with 0.5× the MIC for 30 min prior to post-transcriptional arrest (PTA) with rifampin and *spa* RNA was quantified via RT-PCR at 0- and 5-min PTA, as previously described [9]. In cells that were treated with DMSO, 55% of *spa* transcript was degraded after 5 min PTA, as shown in Figure 8. The RNPA2000 positive control, as well as compounds **7** and **8**, showed a significantly reduced degradation of *spa* transcript. Compound **4** had no effect on cellular RNA turnover, with the treated cells degrading approximately 66% of *spa* after 5 min PTA, despite having in vitro activity. Compound **6**, which also had in vitro activity, resulting in an average of 31% *spa* transcript degradation, an approximate 24% inhibition of degradation when compared to DMSO. Compound **10**, which had weak in vitro degradation activity, had an average inhibition of 22% on cellular RNA turnover in comparison to DMSO.

Taken together, compounds **7** and **8**, presenting a 3-halogenphenyl moiety in place of the 2-furanyl one, performed well when evaluated in in-vitro assays and they were the top performers in both cellular assays, significantly inhibiting both RnpA-mediated cellular RNA metabolism functions. Although compound **10**, in which the *i*-propylphenyloxy portion was replaced by a more lypophilic one, had the most potent antibacterial activity, it had weak effects on RNA degradation (both in vitro and in vivo), but it was a good inhibitor of ptRNA^Tyr^ processing in vitro and it had significantly increased ptRNA^Tyr^ accumulation in cellular assays.

## 3. Conclusions

In the present work, we predicted key interactions that RNPA2000 and JC1/2 achieve with their molecular target RnpA. This information guided the design of new inhibitors, which increased the knowledge of the SAR of RNPA2000-like and JC1/2-like RnpA inhibitors. The lower activity of compounds **1** to **5** as compared to **JC1/2** suggest that the combination of a small-aliphatic amine with a 3,5-dichlorophenyl moiety is required for RnpA inhibition. Nevertheless, further investigation regarding this class of compounds remains worthwhile. Compounds **7** and **8,** characterized by a 3-halogenphenyl ring in place of the 2-furanyl moiety, demonstrated cellular activity that was comparable to **RNPA2000**, while avoiding the potentially metabotoxic furan. At the same time, the inactivity of compounds **11**, **12**, **13**, and **14** underline the importance of the semi-thiocarbazide. This preliminary set of data, which are perfectly in line with the innovative computational model, trace the path for a deeper investigation of the SAR of these very promising classes of compounds. Moreover, an exciting aspect of the current work is the development of a putative computational-based path for RnpA inhibitor discovery and optimization. Building from our early modeling success is expected to allow for the rational design of more potent and presumably more selective molecules that, as they become available and evaluated for RnpA inhibitor properties, are expected to allow refining the computational modeling in iterative manner and yielding higher quality probes for mutagenesis based testing of the model. 

## 4. Materials and Methods

### 4.1. Chemistry

All of the reagents were purchased from Sigma Aldrich (St. Louis, MO, USA) and they were used without further purification. The solvents, such as ACN, THF, DCM, DMF, methanol, and acetone, were purchased from Sigma Aldrich.

Silica gel matrix, with fluorescent indicator 254 nm, was used in analytical thin-layer chromatography (TLC on aluminum foils), and silica gel (particle size 40–63 µm, Merck) was used in flash chromatography on Sepachrom Puriflash XS 420. Visualizations were accomplished with UV light (λ 254 or 280 nm).

The ^1^H-NMR spectra were measured by Varian Mercury 300 NMR spectrometer/Oxford Narrow Bore superconducting magnet operating at 300 MHz. The ^13^C-NMR spectra were acquired operating at 75 MHz. Chemical shifts (δ) are reported in ppm relative to residual solvent as the internal standard. Signal multiplicity is used according to the following abbreviations: s = singlet, d = doublet, dd = doublet of doublets, t = triplet, q = quadruplet, dq = doublet of quadruplets, m = multiplet, bs = broad singlet, and set = septuplet. The final products, **1**–**14**, were analyzed by reverse-phase HPLC using a Waters XBridge C-18 column (5 μm, 4.6 mm × 150 mm) on an Elite LaChrom HPLC system with a diode array detector, with different methods (Table 3). Their purity was quantified at peculiar λ max, depending on each sample, and it resulted to be >95%. The relative retention times are reported in each experimental section. The melting points were determined by DSC analysis over a TA Instruments DSC 1020 apparatus. The ^1^H- and ^13^C-NMR spectra of compounds **1**–**14**, together with their HPLC profiles, are included in the supplementary material S1.

#### Synthesis

*1-(4-Biphenyl)-3-(2-methylpiperdin-1-yl)urea* (**1**): diphenyl phosphoryl azide (DPPA) (2.71 mL, 12.10 mmol) was added dropwise to a solution of biphenyl-4-carboxylic acid (2.00 g, 10.09 mmol) and triethylamine (TEA) (1.69 mL, 12.10 mmol) in toluene (20 mL). The reaction mixture was stirred at RT for 20 min, heated at 95 °C, and then stirred again for 20 min. The mixture was cooled at RT, added with 2-methylpiperidine (1.25 mL, 10.59 mmol), heated again at 95 °C and stirred overnight. Then, the mixture was diluted with ethyl acetate (30 mL), washed twice with 10% aqueous NaHCO_3_, dried over Na_2_SO_4_, filtered, and concentrated under vacuum to give a residue, which was purified by flash chromatography. Elution with cyclohexane/ethyl acetate 80/20 and subsequent crystallization from ethanol (15 vol) gave 0.85 g of **1** as a white solid. Yield: 29% M.p: 164.21 °C. Tr (HPLC): 12.65 min. (Method A), Purity = 99.7%. **^1^H NMR (300 MHz, CDCl_3_,** **δ)** 7.55 (m, 4H), 7.46 (m, 4H), 7.29 (m, 1H), 6.49 (bs, 1H), 4.39 (m, 1H), 3.91 (d, *J* = 12.8 Hz, 1H), 3.02 (t, *J* = 12.8 Hz, 1H), 1.62 (m, 6H), 1.25 (d, *J* = 6.8 Hz, 3H). **^13^C NMR (75 MHz, CDCl_3_,** **δ)** 154.94, 140.76, 138.79, 135.57, 128.68, 127.41, 126.77, 126.71, 120.13, 46.74, 39.13, 30.23, 25.63, 18.55, 15.76 ppm.

*1-(3-Biphenyl)-3-(2-methylpiperdin-1-yl)urea* (**2**): Prepared from biphenyl-3-carboxylic acid as described for **1** using DPPA (1.2 eq), TEA (1.2 eq), and 2-methylpiperdine (1.05 eq) in toluene (10 vol), and then purified by flash chromatography on silica gel. Elution with cyclohexane/ethyl acetate 80/20 and subsequent crystallization from EtOH (4 vol) and recrystallization from IPA (4 vol) gave 0.52 g of **2** as a white solid. Yield: 17% M.p: 126.53 °C. Tr (HPLC): 12.67 min. (Method A), Purity = 99.3%. **^1^H NMR (300 MHz, CDCl_3_,** **δ)** 7.65 (m, 3H), 7.32 (m, 6H), 6.46 (bs, 1H), 4.41 (m, 1H), 3.91 (d, *J* = 13.0 Hz, 1H), 3.02 (t, *J* = 13.0 Hz, 1H), 1.64 (m, 6H), 1.25 (d, *J* = 6.9 Hz, 3H). **^13^C NMR (75 MHz, CDCl_3_,** **δ)** 154.94, 141.91, 140.98, 139.75, 129.16, 128.61, 127.26, 127.18, 121.64, 118.72, 118.68, 46.76, 39.13, 30.23, 25.63, 18.55, 15.77 ppm.

*1-(2-Biphenyl)-3-(2-methylpiperdin-1-yl)urea* (**3**): Prepared from biphenyl-2-carboxylic acid as described for **1** using DPPA (1.2 eq), TEA (1.2 eq). and 2-methylpiperdine (1.05 eq) in toluene (10 vol), and the purified by flash chromatography on silica gel. Elution with cyclohexane/ethyl acetate 80/20 and subsequent crystallization from IPA (4 vol) gave 1.02 g of **3** as a white solid. Yield: 35% M.p: 104.05 °C. Tr (HPLC): 10.67 min. (Method A), Purity = 96.4%. **^1^H NMR (300 MHz, CDCl_3_,** **δ)** δ 8.11 (d, *J* = 8.1 Hz, 1H), 7.41 (m, 6H), 7.20 (dd, *J* = 7.6, 1.6 Hz, 1H), 7.07 (td, *J* = 7.6, 0.9 Hz, 1H), 6.47 (bs, 1H), 3.98 (m, 1H), 3.70 (d, *J* = 12.6 Hz, 1H), 2.78 (t, *J* = 12.6 Hz, 1H), 1.44 (m, 6H), 1.01 (d, *J* = 6.9 Hz, 3H). **^13^C NMR (75 MHz, CDCl_3_,** **δ)** 154.68, 138.72, 136.43, 131.52, 129.52, 129.32, 128.98, 128.38, 127.77, 122.49, 120.85, 46.62, 38.57, 30.19, 25.46, 18.52, 15.58 ppm.

*1-(3,5-Dichlorophenyl)-3-(1,2,3,4-tetrahydroquinolin-1-yl)urea* (**4**): Prepared from 3,5-dichlorobenzoic acid, as described for **1** using DPPA (1.2 eq), TEA (1.2 eq), and 1,2,3,4-tetrahydroquinoline (1.05 eq) in toluene (10 vol) and purified by flash chromatography on silica gel. Elution with cyclohexane/ethyl acetate 80/20 and subsequent crystallization from EtOH (6 vol) gave 0.80 g of **4** as a white solid. Yield: 32% M.p: 143.34 °C. Tr (HPLC): 12.46 min. (Method C), Purity = 99.8%. **^1^H NMR (300 MHz, CDCl_3_,** **δ)** 7.34 (d, *J* = 1.9 Hz, 2H), 7.25 (m, 3H), 7.15 (m, 1H), 7.06 (bs, 1H), 7.00 (t, *J* = 1.8 Hz, 1H), 3.81 (t, *J* = 6.3 Hz, 2H), 2.79 (t, *J* = 6.7 Hz, 2H), 1.98 (m, 2H). **^13^C NMR (75 MHz, CDCl_3_,** **δ)** 152.98, 140.59, 138.25, 135.11, 133.21, 129.89, 127.05, 125.32, 122.88, 122.82, 117.15, 43.43, 26.90, 23.94 ppm.

*1-(3,5-Dichlorophenyl)-3-(1,2,3,4-tetrahydroisoquinolin-1-yl)urea* (**5**): Prepared from 3,5-dichlorobenzoic acid as described for **1** using DPPA (1.2 eq), TEA (1.2 eq), and 1,2,3,4-tetrahydroisoquinoline (1.05 eq) in toluene (10 vol), and then purified by flash chromatography on silica gel. Elution with cyclohexane/ethyl acetate 85/15 and subsequent crystallization from IPE (20 vol) gave 0.60 g of **5** as a white solid. Yield: 24% M.p: 128.79 °C. Tr (HPLC): 9.00 min. (Method C). Purity = 99.9%. **^1^H NMR (300 MHz, CDCl_3_,** **δ)** 7.36 (d, *J* = 1.5 Hz, 2H), 7.18 (m, 4H), 6.98 (s, 1H), 6.75 (bs, 1H), 4.64 (s, 2H), 3.71 (t, *J* = 5.8 Hz, 2H), 2.92 (t, *J* = 5.7 Hz, 2H). **^13^C NMR (75 MHz, CDCl_3_,** **δ)** 154.29, 141.04, 134.92, 134.68, 132.73, 128.39, 126.99, 126.61, 126.29, 122.85, 118.15, 45.77, 41.73, 28.89 ppm.

*Methyl 4-i-propylphenoxyacetate* (**15**): methyl chloroacetate (0.71 mL, 8.01 mmol) was added to a solution of 4-isopropylphenol (1.00 g, 7.34 mmol) and potassium carbonate (1.12 g, 8.01 mmol) in DMF (10 mL). The reaction mixture was stirred at 50 °C for 1.5 h, concentrated under vacuum, diluted with ethyl acetate (30 mL), washed with brine (4 × 10 mL), dried over Na_2_SO_4_, filtered, and then concentrated under vacuum to give 1.4 g of **15** as a yellowish oil. Yield: 92%. **^1^H NMR (300 MHz, CDCl_3_, δ)** 7.14 (d, *J* = 8.7 Hz, 2H), 6.84 (d, *J* = 8.7 Hz, 2H), 4.61 (s, 2H), 3.80 (s, 3H), 2.86 (set, *J* = 6.9 Hz, 1H), 1.22 (d, *J* = 6.9 Hz, 6H).

*4-i-Propylphenoxyacetohydrazide* (**16**): hydrazine hydrate (2.1 mL, 33.60 mmol) was added to a solution of **15** (1.40 g, 6.72 mmol) in methanol (15 mL). The reaction mixture was stirred at reflux for 16 h, concentrated under vacuum, diluted with ethyl acetate (30 mL), washed with brine (10 mL), dried over Na_2_SO_4_, filtered, and concentrated under vacuum to give 1,36 g of **16** as a white solid. Yield = 97%. M.p: 105.21 °C. **^1^H NMR (300 MHz, CDCl_3_, δ):** 7.16 (d, *J* = 8.2 Hz, 2H), 6.83 (d, *J* = 8.2 Hz, 2H), 4.55 (s, 2H), 2.87 (set, *J* = 6.9 Hz, 1H), 1.22 (d, *J* = 6.9 Hz, 6H).

*1-(4-i-Propylphenoxyacetyl)-4-(3,5-dichlorobenzoyl)thiosemicarbazide* (**6**): a solution of 3,5-dichlorobenzoic acid (1.30 g, 6.8 mmol) in SOCl_2_ (6.5 mL) was stirred at reflux for 1 h. Thus, the solution was concentrated under vacuum, diluted with acetonitrile (15 mL), and then added with potassium thiocyanate (1.12 g, 11.56 mmol). The reaction mixture was stirred at RT for 1 h, added with a solution of **16** (1.40 g, 6.8 mmol) in DMF (5 mL), stirred at RT for 30 min, concentrated under vacuum, diluted with ethyl acetate (50 mL), washed with brine (4 × 10 mL), dried over Na_2_SO_4_, filtered, and then concentrated under vacuum to give a solid. Digestion firstly with MeOH (15 vol) at reflux and secondly with ACN (15 vol) at reflux gave 1.3 g of **6** as a yellowish solid. Yield: 43%. M.p: 195.69 °C. Tr (HPLC): 17.60 min. (Method C), Purity = 99.6%. **^1^H NMR (300 MHz, DMSO-*d*_6_, δ)** 12.38 (bs, 1H), 11.95 (bs, 1H), 11.00 (bs, 1H), 7.96 (d, *J* = 1.8 Hz, 2H), 7.89 (t, *J* = 1.8 Hz, 1H), 7.15 (d, *J* = 8.6 Hz, 2H), 6.91 (d, *J* = 8.6 Hz, 2H), 4.68 (s, 2H), 2.82 (set, *J* = 6.9 Hz, 1H), 1.16 (d, *J* = 6.9 Hz, 6H). **^13^C NMR (75 MHz, DMSO-*d*_6_, δ)** 177.66, 165.66, 165.61, 156.23, 141.70, 135.56, 134.63, 132.58, 127.88, 127.55, 115.02, 66.24, 33.06, 24.50 ppm.

*1-(4-i-Propylphenoxyacetyl)-4-(3-chlorobenzoyl)thiosemicarbazide* (**7**): Prepared from 3-chlorobenzoic acid, as described for **6** using SOCl_2_ (5 vol), potassium thiocyanate (1.6 eq) and **16** (1 eq) in acetonitrile (10 vol) at RT for 30 min. Digestion of the crude product with IPA (10 vol) at reflux gave 1.06 g of **7** as a pale yellow solid. Yield: 27%. M.p: 187.27 °C. Tr (HPLC): 9.38 min. (Method C), Purity = 98.5%. **^1^H NMR (300 MHz, DMSO-*d*_6_, δ)** 12.47 (bs, 1H), 11.90 (bs, 1H), 11.01 (bs, 1H), 8.00 (t, *J* = 1.9 Hz, 1H), 7.86 (m, 1H), 7.70 (ddd, *J* = 8.0, 1.9, 1.0 Hz, 1H), 7.54 (t, *J* = 8.0 Hz, 1H), 7.15 (d, *J* = 8.7 Hz, 2H), 6.91 (d, *J* = 8.7 Hz, 2H), 4.68 (s, 2H), 2.82 (set, *J* = 6.9 Hz, 1H), 1.15 (d, *J* = 6.9 Hz, 6H). **^13^C NMR (75 MHz, DMSO-*d*_6_, δ)** 178.09, 167.00, 156.66, 156.24, 141.69, 134.31, 133.56, 133.25, 130.81, 128.92, 127.91, 127.59, 115.00, 66.16, 33.05, 24.54 ppm.

*1-(4-i-Propylphenoxyacetyl)-4-(3-bromobenzoyl)thiosemicarbazide* (**8**): Prepared from 3-bromobenzoic acid as described for **6** using SOCl_2_ (5 vol), potassium thiocyanate (1.6 eq) and **16** (1 eq) in acetonitrile (10 vol) at RT for 30 min. Digestion of the crude product with IPA (5 vol) at reflux gave 1.96 g of **8** as a white solid. Yield: 62%. M.p: 184.01 °C. Tr (HPLC): 10.13 min. (Method C), Purity = 98.5%. **^1^H NMR (300 MHz, DMSO-*d*_6_, δ)** 12.46 (bs, 1H), 11.90 (bs, 1H), 11.03 (bs, 1H), 8.14 (t, *J* = 1.7 Hz, 1H), 7.92 (d, *J* = 7.9 Hz, 1H), 7.83 (m, 1H), 7.47 (t, *J* = 7.9 Hz, 1H), 7.15 (d, *J* = 8.7 Hz, 2H), 6.91 (d, *J* = 8.7 Hz, 2H), 4.68 (s, 2H), 2.81 (set, *J* = 6.9 Hz, 1H), 1.15 (d, *J* = 6.9 Hz, 6H). **^13^C NMR (75 MHz, DMSO-*d*_6_, δ)** 178.13, 166.89, 165.67, 156.24, 141.69, 136.13, 134.50, 131.73, 131.04, 128.28, 127.59, 121.92, 114.99, 66.15, 33.05, 24.54 ppm.

*1-(4-i-Propylphenoxyacetyl)-4-(3,5-dibromobenzoyl)thiosemicarbazide* (**9**): prepared from 3,5-dibromobenzoic acid, as described for **6** using SOCl_2_ (5 vol), potassium thiocyanate (1.6 eq), and **16** (1 eq) in acetonitrile (10 vol) at RT for 30 min. Digestion of the crude product firstly with MeOH (10 vol) and secondly with IPA (15 vol) at reflux gave 2.00 g of **9** as a white solid. Yield: 62%. M.p: 201.83 °C. Tr (HPLC): 6.19 min. (Method D), Purity = 99.0%. **^1^H NMR (300 MHz, DMSO-*d*_6_, δ)** 12.36 (bs, 1H), 11.97 (bs, 1H), 11.03 (bs, 1H), 8.11 (s, 3H), 7.15 (d, *J* = 8.3 Hz, 2H), 6.87 (dd, *J* = 8.3 Hz, 2H), 4.67 (s, 2H), 2.82 (set, *J* = 6.9 Hz, 1H), 1.15 (d, *J* = 6.9 Hz, 7H). **^13^C NMR (75 MHz, DMSO-*d*_6_, δ)** 177.89, 165.68, 165.48, 156.24, 141.68, 137.88, 136.01, 131.04, 131.04, 127.59, 122.81, 114.99, 66.14, 33.05, 24.54 ppm.

*Methyl 4-biphenyloxyacetate* (**17**): prepared from 4-hydoxybiphenyl as described for **15** using methyl 2-chloroacetate (1.25 eq) and potassium carbonate (1.2 eq) in DMF (10 vol) to give 2.85 g of **17** as a white solid. Yield: 93%. M.p: 100.98 °C. **^1^H NMR (300 MHz, CDCl_3_, δ)** 7.53 (m, 4H), 7.41 (m, 2H), 7.31 (m, 1H), 6.99 (d, *J* = 8.8 Hz, 2H), 4.68 (s, 2H), 3.83 (s, 3H).

*4-Biphenyloxyacetohydrazide* (**18**): prepared from **17** as described for **16** using hydrazine hydrate (5 eq) in MeOH (30 mL) to give 1,85 g of **18** as a white solid. Yield: 64%. M.p: 112.86 °C. **^1^H NMR (300 MHz, DMSO-*d*_6_, δ):** δ 9.43 (bs, 1H), 7.58 (m, 4H), 7.41 (t, *J* = 7.4 Hz, 2H), 7.29 (t, *J* = 7.4 Hz, 1H), 7.02 (dd, *J* = 9.3, 2.4 Hz, 2H), 4.53 (s, 2H), 4.33 (s, 2H).

*1-(4-Biphenyloxyacetyl)-4-(3,5-dichlorobenzoyl)thiosemicarbazide* (**10**): prepared from 3,5-diclorobenzoic acid as described for **6** using SOCl_2_ (5 vol), potassium thiocyanate (1.6 eq) and **18** (1 eq) in acetonitrile (10 vol) at RT for 1 h. Digestion of the crude product with MeOH (10 vol) at reflux gave 2.77 g of **10** as a white solid. Yield: 73%. M.p: 200.71 °C. Tr (HPLC): 13.81 min. (Method C), Purity = 95.2%. **^1^H NMR (300 MHz, DMSO-*d*_6_, δ)** 12.37 (bs, 1H), 11.98 (bs, 1H), 11.12 (bs, 1H), 7.96 (d, *J* = 1.9 Hz, 2H), 7.89 (t, *J* = 1.9 Hz, 1H), 7.59 (m, 4H), 7.42 (t, *J* = 7.6 Hz, 2H), 7.33–7.25 (m, 1H), 7.08 (d, *J* = 8.8 Hz, 2H), 4.77 (s, 2H). **^13^C NMR (75 MHz, DMSO-*d*_6_, δ)** 178.03, 165.63, 165.56, 157.78, 140.12, 135.70, 134.58, 133.79, 132.60, 129.31, 128.20, 127.92, 127.27, 126.69, 115.64, 66.11 ppm.

*4-i-Propylphenoxyacetic acid* (**19**): 2.5 N aqueous NaOH (6 mL, 15 mmol) was added to a solution of **15** (3.00 g, 14.40 mmol) in MeOH (30 mL). The reaction mixture was stirred at RT for 1.5 h, concentrated under vacuum, diluted with water, acidified to pH = 1 with conc. HCl, extracted twice with ethyl acetate (2 × 30 mL), dried over Na_2_SO_4_, filtered, and concentrated under vacuum to give 2.60 g of **19** as a white solid. Yield: 95%. M.p: 82.14 °C. **^1^H NMR (300 MHz, CDCl_3_, δ)** 7.16 (d, *J* = 8.6 Hz, 2H), 6.86 (d, *J* = 8.6 Hz, 2H), 4.66 (s, 2H), 2.86 (set, *J* = 6.9 Hz, 1H), 1.22 (d, *J* = 6.9 Hz, 6H). 

*4-i-Propylphenoxyacetyl chloride* (**20**): SOCl_2_ (10 mL, 5 vol) was added to **19** (2.00 g, 10.30 mmol). The reaction mixture was stirred at reflux for 1 h and concentrated under vacuum to give 2.15 g of **20** as a dark oil. Yield: 98%. **^1^H NMR (300 MHz, CDCl_3_, δ)** 7.17 (d, *J* = 8.6 Hz, 2H), 6.83 (d, *J* = 8.6 Hz, 2H), 4.92 (s, 2H), 2.87 (set, *J* = 6.9 Hz, 1H), 1.22 (d, *J* = 6.9 Hz, 6H).

*N-(t-Butoxycarbonylaminomethyl)-4-i-propylphenoxyacetamide* (**21**): A solution of *N*-Boc methylendiamine (1.20 g, 8.21 mmol) in DCM (5 mL) was added to a solution of **20** (1.6 g, 8.21 mmol) and TEA (1.37 mL, 9.85 mmol) in DCM (5 mL) at 0 °C. The reaction mixture was stirred at RT for 3 h, diluted with DCM (20 mL), washed twice with 10% aqueous NaHCO_3_ (2 × 10 mL) and once with brine (10 mL), dried over Na_2_SO_4_, filtered and concentrated under vacuum to give 2.17 g of **21** as a yellowish oil. **^1^H NMR (300 MHz, CDCl_3_, δ)** 7.50 (bs, 1H), 7.15 (d, *J* = 8.6 Hz, 2H), 6.83 (d, *J* = 8.6 Hz, 2H), 5.57 (bs, 1H), 4.62 (t, *J* = 6.4 Hz, 2H), 4.45 (s, 2H), 2.86 (set, *J* = 6.9 Hz, 1H), 1.43 (s, 9H), 1.22 (d, *J* = 6.9 Hz, 6H).

*N-(Aminomethyl)-2-(4-i-propylphenoxy)acetamide* (**22**): 10% aqueous HCl (2.5 mL, 7.5 mmol) was added to a solution of **21** (1.00 g, 3.10 mmol) in MeOH (10 mL) at RT. The reaction mixture was stirred at reflux for 0.5 h, concentrated under vacuum, diluted with Et_2_O (30 mL) and extracted twice with 10% aqueous HCl (2 × 15 mL). The aqueous phase was thus basified to pH = 12 and extracted three times with ethyl acetate (3 × 25 mL), dried over Na_2_SO_4_, filtered and concentrated under vacuum to give 0.40 g of **22** as a yellowish oil. Yield: 58%. **^1^H NMR (300 MHz, CDCl_3_,** **δ)** 7.26 (bs, 1H), 7.16 (d, *J* = 8.4 Hz, 1H), 6.84 (d, *J* = 8.4 Hz, 1H), 4.46 (s, *J* = 20.8 Hz, 2H), 4.30 (s, 2H), 2.86 (set, *J* = 6.9 Hz, 1H), 2.36 (bs, 2H), 1.24 (dd, *J* = 14.8, 7.0 Hz, 6H).

*3,5-Dichloro-N-((2-(4-i-propylphenoxy)acetamido)methyl)benzamide* (**11**): prepared from **22** as described for **21** using 3,5-dichlorobenzoyl chloride (1 eq), TEA (1.2 eq) in DCM (20 mL) at RT for 1.5 h. Digestion of the crude product with IPA (10 vol) at reflux gave 0.85 g of **11** as a white solid. Yield: 70%. M.p: 189.88 °C. Tr (HPLC): 9.94 min. (Method B), Purity = 97.0%. **^1^H NMR (300 MHz, DMSO-*d*_6_, δ)** 9.30 (t, *J* = 5.4 Hz, 1H), 8.63 (t, *J* = 5.4 Hz, 1H), 7.88 (d, *J* = 1.7 Hz, 2H), 7.80 (s, 1H), 7.10 (d, *J* = 8.6 Hz, 2H), 6.83 (d, *J* = 8.6 Hz, 2H), 4.68 (t, *J* = 5.4 Hz, 2H), 4.46 (s, 2H), 2.79 (set, *J* = 6.9 Hz, 1H), 1.12 (d, *J* = 6.9 Hz, 6H).**^13^C NMR (75 MHz, DMSO-*d*_6_, δ)** 168.74, 164.30, 156.20, 141.50, 137.40, 134.73, 131.32, 127.51, 126.69, 114.91, 67.21, 44.63, 33.01, 24.49 ppm.

*N-(t-Butoxycarbonylaminoethyl)-3,5-dichlorobenzamide* (**23**): prepared from 3,5-dichlorobenzoyl chloride as described for **21** using *N*-boc ethylendiamine (1.5 eq) and TEA (1.5 eq) in DCM (10 vol) to give 2.85 g of **23** as a pale yellow oil. Yield: 81%. **^1^H NMR (300 MHz, CDCl_3_, δ)** 7.71 (d, *J* = 1.7 Hz, 2H), 7.57 (bs, 1H), 7.45 (t, *J* = 1.7 Hz, 1H), 5.07 (bs, 1H), 3.52 (m, 2H), 3.40 (m, 2H), 1.44 (s, 9H).

*N-(2-Aminoethyl)-3,5-dichlorobenzamide* (**24**): prepared from **23** as described for **22** using 10% aqueous HCl (2 eq) in MeOH (10 vol) to give 1.61 g of **24** as a brownish oil. Yield: 80%. **^1^H NMR (300 MHz, CDCl_3_,** **δ)** 7.64 (d, *J* = 1.6 Hz, 2H), 7.43 (t, *J* = 1.6 Hz, 1H), 7.14 (bs), 3.46 (dd, *J* = 10.9, 5.4 Hz, 2H), 2.93 (t, *J* = 5.7 Hz, 2H), 1.67 (bs, 2H).

*3,5-Dichloro-N-((2-(4-i-propylphenoxy)acetamido)ethyl)benzamide* (**12**): prepared from **24** as described for **21** using **20** (1 eq), TEA (1.2 eq) in DCM (20 mL) at RT for 1 h. Digestion of the crude product with IPA (10 vol) at reflux gave 1.68 g of **12** as a white solid. Yield: 59%. M.p: 181.73 °C. Tr (HPLC): 7.21 min. (Method C), Purity = 97.1%. **^1^H NMR (300 MHz, DMSO-*d*_6_, δ)** 8.70 (t, *J* = 4.6 Hz, 1H), 8.16 (t, *J* = 4.6 Hz, 1H), 7.82 (d, *J* = 1.7 Hz, 2H), 7.78 (t, *J* = 1.7 Hz, 1H), 7.09 (d, *J* = 8.6 Hz, 2H), 6.84 (d, *J* = 8.6 Hz, 2H), 4.40 (s, 2H), 3.33 (m, 4H), 2.77 (sec, *J* = 6.9 Hz, 1H), 1.12 (d, *J* = 6.9 Hz, 6H). **^13^C NMR (75 MHz, DMSO-*d*_6_, δ)** 168.87, 164.17, 156.31, 141.52, 138.12, 134.65, 130.89, 127.41, 126.52, 115.01, 67.70, 38.51, 33.02, 24.44 ppm.

*4-t-Butoxycarbonylamino-1-(4-i-propylphenoxyacetyl)piperidine* (**25**): prepared from **20** as described for **21** using 4-*N*-Boc-aminopiperidine (1 eq), TEA (1.2 eq) in DCM (20 mL) at RT for 3 h. Crystallization of the crude product with MeOH (7.5 vol) gave 2.80 g of **25** as a white solid. Yield: 60%. M.p: 160,53 °C **^1^H NMR (300 MHz, CDCl_3_, δ)** 7.13 (d, *J* = 8.5 Hz, 2H), 6.85 (d, *J* = 8.5 Hz, 2H), 4.63 (s, 1H), 4.44 (m, 2H), 3.95 (d, *J* = 13.1 Hz, 1H), 3.66 (bs, 1H), 3.13 (t, *J* = 12.2 Hz, 1H), 2.83 (m, 1H), 2.82 (set, *J* = 6.9 Hz, 1H) 1.97 (t, *J* = 12.5 Hz, 2H), 1.43 (s, 9H), 1.20 (d, *J* = 6.9 Hz, 6H).

*4-Amino-1-(4-i-propylphenoxyacetyl)piperidine* (**27**): TFA (5.66 mL, 73.80 mmol) was added to a solution of **25** (2.78 g, 7.38 mmol) in DCM (30 mL) at 0 °C. The reaction mixture was stirred at RT for 18 h, diluted with DCM, washed once with 10% aqueous NaOH (15 mL), once with brine (15 mL), dried over Na_2_SO_4_, filtered, and concentrated under vacuum to give 2.04 g of **27** as a yellowish oil. Yield: Quantitative. **^1^H NMR (300 MHz, CDCl_3_, δ)** 7.13 (d, *J* = 8.6 Hz, 2H), 6.86 (d, *J* = 8.6 Hz, 2H), 4.64 (s, 2H), 4.44 (d, *J* = 13.6 Hz, 1H), 3.96 (d, *J* = 13.6 Hz, 1H), 3.10 (t, *J* = 12.4 Hz, 1H), 2.85 (m, 2H), 2.82 (set, *J* = 6.9 Hz, 1H), 1.84 (m, 2H), 1.30 (m, 2H), 1.21 (d, *J* = 6.9 Hz, 6H).

*4-(3,5-Dichlorobenzoylamino)-1-(4-i-propylphenoxyacetyl)piperidine* (**13**): prepared from **27** as described for **21** using 3,5-dichlorobenzoyl chloride (1 eq), TEA (1.2 eq) in DCM (20 mL) at RT for 3 h. Crystallization of the crude product with MeOH (5 vol) at reflux gave 0.81 g of **13** as a white solid. Yield: 43%. M.p: 135.90 °C. Tr (HPLC): 14.83 min. (Method B), Purity = 98.4%. **^1^H NMR (300 MHz, DMSO-*d*_6_, δ)** 8.53 (d, *J* = 7.5 Hz, 1H), 7.86 (m, 2H), 7.78 (m, 1H), 7.12 (d, *J* = 8.2 Hz, 2H), 6.83 (d, *J* = 8.2 Hz, 2H), 4.79 (d, *J* = 14.0 Hz, 1H), 4.71 (d, *J* = 14.0 Hz, 1H), 4.28 (d, *J* = 13.1 Hz, 1H), 4.02 (m, 1H), 3.86 (d, *J* = 13.1 Hz, 1H), 3.15 (t, *J* = 12.1 Hz, 1H), 2.82 (set, *J* = 6,8 Hz, 1H), 2.80 (m, 1H), 1.85 (m, 2H), 1.53 (m, 1H), 1.37 (m, 1H), 1.15 (d, *J* = 6.8 Hz, 6H). **^13^C NMR (75 MHz, DMSO-*d*_6_, δ)** 166.23, 163.25, 156.59, 141.21, 138.11, 134.66, 131.00, 127.47, 126.59, 114.79, 66.69, 47.27, 43.69, 33.03, 32.16, 31.40, 24.55 ppm.

*4-t-Butoxycarbonyl-1-(4-i-propylphenoxyacetyl)piperazine* (**26**): Prepared from **20** as described for **21** using *N*-Boc-piperazine (1 eq), TEA (1.2 eq) in DCM (20 mL) at RT for 3 h to give 1.86 g of **26** as a yellow oil. Yield: Quantitative. **^1^H NMR (300 MHz, CDCl_3_, δ)** 7.14 (d, *J* = 7.1 Hz, 2H), 6.86 (d, *J* = 7.1 Hz, 2H), 4.66 (s, 2H), 3.57 (m, 4H), 3.43 (m, 4H), 2.85 (set, *J* = 6.8 Hz, 1H), 1.45 (s, 9H), 1.21 (d, *J* = 6.8 Hz, 6H).

*1-(4-i-Propylphenoxyacetyl)piperazine* (**28**): prepared from **26** as described for **27** using TFA (10 eq) in DCM (10 vol) at RT for 18 h to give 1.35 g of **28** as a yellowish oil. Yield: Quantitative. **^1^H NMR (300 MHz, CDCl_3_,** **δ)** 7.14 (d, *J* = 8.7 Hz, 2H), 6.87 (d, *J* = 8.7 Hz, 2H), 4.65 (s, 2H), 3.59 (m, 4H), 2.85 (m, 4H), 2.82 (set, *J* = 7.0 Hz, 1H) 1.22 (d, *J* = 7.0 Hz, 6H).

*4-(3,5-Dichlorobenzoyl)-1-(4-i-propylphenoxyacetyl)piperazine* (**14**): prepared from **28** as described for **21** using 3,5-dichlorobenzoyl chloride (1 eq), TEA (1.2 eq) in DCM (20 mL) at RT for 3 h. Crystallization of the crude product with 7/3 cyclohexane/ethyl acetate (5 vol total) at reflux gave 0.95 g of **14** as a white solid. Yield: 42%. M.p: 104.08 °C. Tr (HPLC): 6.67 min. (Method C), Purity = 98.0%. **^1^H NMR (300 MHz, DMSO-*d*_6_, δ)** 7.72 (m, *J* = 1.7 Hz, 1H), 7.50 (m, 2H), 7.12 (d, *J* = 8.1 Hz, 2H), 6.82 (d, *J* = 8.1 Hz, 2H), 4.78 (s, 2H), 3.44 (m, 8H), 2.80 (set, *J* = 6.9 Hz, 1H), 1.14 (d, *J* = 6.9 Hz, 6H). **^13^C NMR (75 MHz, DMSO-*d*_6_, δ)** 166.70, 166.62, 156.48, 141.27, 139.65, 134.76, 129.58, 127.47, 126.09, 114.85, 66.49, 33.02, 24.55 ppm.

### 4.2. Biological Evaluation

#### 4.2.1. Bacterial Growth Conditions

*Staphylococcus aureus* strains UAMS-1 is a well-characterized antibiotic-susceptible osteomyleitus clinical isolate (Blevins Infect. Immun 2003). It was cultured in Mueller Hinton (MH) broth and processed. as described below. *Escherichia coli* strain BL21 (DE3) was cultured in Luria–Bertani (LB) and supplemented with ampicillin (50 µg mL^−1^).

#### 4.2.2. Antimicrobial Susceptibility Testing

##### ATCC 29213 and ATCC 43300

The antibacterial activity was tested on both a methicillin-sensitive *S. aureus strain* (MSSA, ATCC 29213) and a methicillin-resistant *S. aureus* strain (MRSA, ATCC 43300). All of the compounds were dissolved in dimethyl sulphoxide (DMSO). Fresh cell cultures were used at 10^5^ cells/mL in a final volume of 2 mL. Each bacterial sample was grown with different compound concentrations at 37 °C for 16 h in aerobic culture tubes. The concentration of bacterial cells was then determined by optical density measurement, at 600 nm (OD_600_) in a SmartSpec^TM^ 3000 spectrophotometer (Bio-Rad, Oceanside, CA, USA). The presence of antibacterial activity, at any concentration tested, was established for values of absorbance <0.1 OD_600_. The MIC was determined as the lowest compound concentration at which the bacterial cell growth was inhibited.

All of the tests were performed in quadruplicate and, for each series of experiments, both positive (bacterial cells cultures with no compound) and negative (fresh medium tubes with compound in the absence of bacteria) controls were included.

#### 4.2.3. RnpA Protein Purification

His-tagged RnpA was purified, as previously described (Eidem AAC 2015). *E. coli* BL21 (DE3) cells harboring plasmid pEXP5-nt containing a hexahistidine tag fused to the *N*-terminus of *S. aureus* RnpA coding region were grown in LB supplemented with 50 µg mL^−1^ ampicillin to OD_600_ = 0.6 and then induced with 1 mM isopropyl β-d-1-Thiogalactopyranoside (IPTG) for 3 h to induce protein expression. *E. coli* cells were subjected to centrifugation at 3.488× *g* for 20 min at 4 °C and the cell pellet was resuspended in 20 mL buffer A (300 mM NaCl, 50 mM Na_2_HPO_4_, pH 7.4) containing a mini EDTA-free protease inhibitor tablet (Roche; Bradford, CT, USA) and 20 mM imidazole. Cells were mechanically lysed by three passes at 18,000 psi through a French Pressure Cell Press (SLM-Aminco; Pittsford, NY, USA) and cell debris was removed by centrifugation at 17,000× *g* for 10 min at 4 °C. Supernatants were collected, filtered through a 0.2 µM syringe filter, and then loaded onto a 5 mL HisPur cobalt column (ThermoFisher Scientific) using the BioRad Maximizer Duo-Flow Medium Pressure Chromatography System. The protein was eluted using an imidazole gradient (80 mM to 500 mM). Fractions were assessed for RnpA presence and purity via SDS-PAGE analysis, Coomassie staining, and Western blotting using an anti-His antibody (Invitrogen; Grand Island, NY, USA). His-RnpA was eluted off the column at imidazole concentrations ranging between 200–300 mM. Fractions that only contained bands corresponding to His-RnpA were pooled and stored at 4 °C. No additional dialysis or concentration protocols were implemented. ptRNA processing assays were performed with RnpA and ptRNA substrate in the absence of *rnpB*, as controls to eliminate the possibility that the protein preparation contained undetected contaminating RNase proteins. Because ptRNA processing requires the RNase P enzyme (RnpA+*rnpB*), no processing activity was detected with RnpA alone, which confirmed the absence of contaminating RNases (data not shown).

#### 4.2.4. In-Vitro Transcription of RNA

*RnpB* and RNA substrates (*spa*, ptRNA^Tyr^) for RnpA functional assays, as well as mature tRNA, were synthesized in vitro, as previously described (Eidem AAC 2015). Each gene was PCR amplified using *S. aureus* UAMS-1 chromosomal DNA as a template and the corresponding oligonucleotide primer pairs, in which the forward primers contained a RNA polymerase T7 promoter sequence. The following primer pairs were used in this study (T7 promoter underlined): T7-*rnpB* forward—GAT TAC ATA ATA CGA CTC ACT ATA GGG TGA TAT TTC GGG TAA TCG CTA TA and *rnpB* reverse—ACT AGT AGT GAT ATT TCT ATA AGC CAT G; T7-ptRNA^Tyr^ forward—GAT TAC ATA A TA CGA CTC ACT ATA GGG CAC CAT TTA TGG AGG GGT AGC G and ptRNA^Tyr^ reverse—TGGTGGAG GGGGGCAGATTC; T7-*spa* forward—GAT TAC ATA ATA CGA CTC ACT ATA GGG TTA TAG TTC GCG ACG ACG TCC AG and *spa* reverse—TTG AAA AAG AAA AAC ATT TAT TCA ATT CGT AAA CTA GG. The resulting PCR products were analyzed on a 0.8% agarose gel and then purified using the Qiagen QIAquick Gel Extraction kit, according to the manufacturer’s instructions. In-vitro transcription was performed using the TranscriptAid T7 High Yield Transcription kit (Fermentas; Burlington, ON, Canada) following the manufacturer’s instructions. Following in-vitro transcription, the RNA was treated with DNaseI for 90 min and then re-purified using the Qiagen RNeasy Mini kit. RNA was quantified using a NanoDrop2000 spectrophotometer.

#### 4.2.5. In-Vitro ptRNA Processing Assays

*S. aureus* RNase P activity assays were performed as previously described (Eidem AAC 2015). Briefly, ptRNA^Tyr^ and *rnpB* RNA was denatured by heating at 95 °C for 5 min and then slowly cooled to RT. RNA species were then combined with 2 × low salt buffer (50 mM Tris-HCl pH 8.0, 5 mM MgCl_2_) and incubated for 5 min at 37 °C. RNase P was reconstituted by mixing an equal molar ratio of His-RnpA and *rnpB* for 15 min at 37 °C. Precursor tRNA processing inhibition reactions were performed by combining 5 pmol of reconstituted RNase P with DMSO (negative control) or compound and incubating for 5 min at 37 °C. 5 pmol ptRNA^Tyr^ was added to each reaction and incubated for an additional 30 min at 37 °C. Reactions were stopped with 2 × RNA loading dye (Thermo Scientific) and heating at 65 °C for 10 min. The samples were electrophoresed on a 7 M urea/8% polyacrylamide gel and then stained with 0.5 µg mL^−1^ ethidium bromide. The BioRad EZ Gel Doc imaging system was used to visualize the RNA and the relative abundance of mature tRNA^Tyr^ in the DMSO control or in the samples containing the compound. Samples were analyzed using the BioRad Image Lab densitometry software. The percent inhibitory activity of each compound was calculated using the following equation: (% experimental processing/% processing negative control) × 100.

#### 4.2.6. In-Vitro mRNA Degradation Assays

RnpA-mediated degradation was assessed using the RNase Alert QC System (Thermofisher Scientific). His-RnpA (20 pmol of was combined with 0.4 μM (final concentration) FRET substrate and either DMSO (negative control) or compound (≤500 µM in 1 × RNase Alert Buffer). Reactions were assembled in 96-well PCR plates (BioRad) and incubated at 37 °C for 30 min in the BioRad CFX 96 Connect Real Time instrument, measuring fluorescence every 2 min at 490 nm excitation/520 nm emission. Percent inhibition of each compound was calculated at the 30 min time-point based on the following formula: (RFU of compound treated RnpA/RFU of DMSO-treated RnpA) × 100.

#### 4.2.7. Cellular tRNA^Tyr^ Population Measures

Cellular *S. aureus* tRNA^Tyr^ pools were measured, as previously described (Colquhoun Antibiotics 2019). Briefly, *S. aureus* was grown to OD_600_ = 0.18 in MHB and then treated with DMSO (negative control), 0.5× or 1× MIC of the indicated compound for 1 h with shaking at 37 °C. The culture was removed, combined with an equal volume of acetone:ethanol (1:1 *v*/*v*), and stored at −80 °C.

#### 4.2.8. Cellular mRNA Turnover Assays

*S. aureus* RNA half-life determinations were performed, as described (Eidem AAC 2015). Briefly, *S. aureus* UAMS-1 was grown to an optical density of 0.18 at 600_nm_ in MHB and then treated with DMSO (negative control) or 0.5× MIC of the indicated compound for 30 min with shaking at 37 °C. To inhibit *de novo* RNA synthesis, rifampin (Alfa Aesar, Haverhill, MA, USA) was added to the culture at a final concentration of 200 µg mL^−1^. Half of the culture was immediately removed and combined with an equal volume of ice-cold acetone:ethanol (1:1 *v*/*v*). The remainder of the culture was returned to the 37 °C incubator for an additional 5 min and then combined with an equal volume of ice-cold acetone: ethanol (1:1 *v*/*v*) and stored at −80 °C.

#### 4.2.9. Bacterial RNA Isolation and Quantitative Reverse Transcription Polymerase Chain Reaction (qRT-PCR)

Cell suspensions in acetone:ethanol were thawed on ice and centrifuged at 1560× *g* for 10 min at 4 °C. Acetone:ethanol was decanted and the pellets were air-dried for 5 min Cell pellets were resuspended in 500 µL ice-cold TE buffer (10 mM Tris-HCl, pH 8.0, 1 mM EDTA) and transferred to FastPrep Lysing Matrix B tubes (MP Biomedicals, Santa Ana, CA, USA). Mixtures were homogenized at 5 m∙s^−1^ for 20 s, rested on ice for 5 min, then homogenized again at 4.5 m∙s^−1^ for 20 s in a FastPrep-24 instrument. Cell lysates were centrifuged at 16,200× *g* for 15 min at 4 °C to remove cell debris. The total bacterial RNA and tRNA^Tyr^ populations were isolated from the supernatant using Qiagen RNeasy Mini kits and miRNeasy kits, respectively, following the manufacturer’s recommendations (Germantown, MD, USA). For qRT-PCR, 2 µg of RNA substrate was treated with two units of DNaseI (New England Biolabs, Ipswich, MA, USA) for 1 h at 37 °C and then re-purified using Qiagen RNeasy Mini kits, following the manufacturer’s instructions. RNA was measured for concentration and quality using a NanoDrop spectrophotometer. Quantabio (Beverly, MA, USA) qScript cDNA Supermix was used to convert 200 ng of RNA into cDNA, following the manufacturer’s instructions, which was subsequently amplified using Quantabio SYBR Green Fast Mix following the manufacturer’s instructions. Fluorescence was read on the BioRad (Hercules, CA, USA) CFX 96 Connect Real Time Machine. The transcript levels were compared to the internal control 16S rRNA (ΔΔCt) and plotted as fold change compared to the control. The following primer pairs were used in this study for qRT-PCR: *spa* forward—GCA GAT AAC AAA TTA GCT GAT AAA AAC AT; *spa* reverse—CTA ACG CTA ATG ATA ATC CAC CAA ATA C; −15 ptRNA^Tyr^ forward—TTA ACT GAA TAA GCT GGA GGG G; tRNA^Tyr^ reverse—TGG TGG AGG GGG GCA GAT TC; 16S rRNA forward—TAA CCT ACC TAT AAG ACT GGG ATA A; 16S rRNA reverse—GCT TTC ACA TCA GAC TTA AAA A.

### 4.3. Hotspot Maps

The Fragment Hotspot maps software [28] identifies the location and environment of binding sites on the protein by first calculating atomic hotspots and then producing Fragment Hotspot maps while using simple molecular probes. These maps specifically highlight fragment-binding sites and their corresponding pharmacophores. For this reason and given the limited knowledge about ligand binding in this target, we applied hotspot maps to decipher the potential binding site for the set of molecules synthesized in this work. For this study, we used the crystal structure of *S. aureus* RNase P protein already available as 6D1R [26] in the Protein Data Bank.

### 4.4. Computational Studies

Automated docking was used to assess the potential binding pocket of the set of molecules together with their binding conformations. A Lamarckian genetic algorithm [29] method that was implemented in the program, AutoDock 4.2 [30], was employed. For docking calculations, Gasteiger charges were added, rotatable bonds were set using AutoDock tools (ADT), and all of the torsions were allowed to rotate for the ligand. As a first approach, blind docking was carried out with the reference compounds RNPA2000 and JC1, including all the receptor as part of the grid to assess the potential binding site of these compounds. For docking the compounds that were synthesized in this work, we used grid maps with a grid box size of 60-by-60-by-60 Å^3^ points and a grid-point spacing of 0.375 Å. The docking protocol consisted of 100 independent Genetic Algorithm (GA) runs, a population size of 200, and a maximum of 250,000 evaluations, while the other parameters were set as default. The final best-docked clusters, within the default 2.0-Å root mean square deviation (RMSD), according to the binding energies and relative population data provided by Autodock, were analyzed by visual inspection.

## Figures and Tables

**Table 1:** Antimicrobial activity of compounds **1**–**14**.

Compound Name	MSSA (ATCC 29213)	MRSA (ATCC 43300)
	MIC (μM)	MIC (μM)
**1**	>500	>500
**2**	>500	>500
**3**	>500	>500
**4**	311	311
**5**	>500	>500
**6**	21.1	21.1
**7**	24.7	24.7
**8**	22.2	22.2
**9**	>500	18.9
**10**	21.1	21.1
**11**	>500	>500
**12**	>500	>500
**13**	>500	>500
**14**	>500	>500

**Table 2:** IC_50_ values of compounds **1**–**14**.

Compound Name	Degradation IC_50_ *	Processing IC_50_ **
RNPA2000	275	140
**1**	72.5	36
**2**	233	37
**3**	324	>500
**4**	66	50
**5**	>500	75
**6**	53	59
**7**	77	28
**8**	49	76
**9**	*-*	*-*
**10**	188	33
**11**	31	153
**12**	165	423
**13**	198	>500
**14**	174	25

* Degradation IC_50_ = in vitro mRNA degradation IC_50_; ** Processing IC_50_ = in vitro ptRNA processing IC_50_.

**Table 3:** HPLC Methods for the analysis of compounds **1**–**14**.

Method Name	Mobile Phase	Flow Rate (mL/min)
A	H_2_O (1‰ TFA)/ACN (1‰ TFA) 55/45	1
B	H_2_O (1‰ TFA)/ACN (1‰ TFA) 55/45	1.5
C	H_2_O (1‰ TFA)/ACN (1‰ TFA) 1/1	1.5
D	H_2_O (1‰ TFA)/ACN (1‰ TFA) 40/60	1.5

## Supplementary Materials

The following are available online at https://www.mdpi.com/article/10.3390/antibiotics10040438/s1, File S1: Supporting Information.
