# Supplementary material for: Staphylococcus aureus RnpA Inhibitors: Computational-Guided Design, Synthesis and Initial Biological Evaluation
Source: Antibiotics (Basel). 2021 Apr 14;10(4):438. doi: 10.3390/antibiotics10040438 (PMC8071009; doi:10.3390/antibiotics10040438)
Supplement: Supplementary file 1 [file antibiotics-10-00438-s001.zip › SupportingInformation_Antibiotics_Suigo_2021.pdf]

Supporting Information

"*Staphylococcus aureus* RnpA Inhibitors: Computational-Guided Design, Synthesis and Initial Biological Evaluation"

Lorenzo Suigo, Michaelle Chojnacki, Carlo Zanotto, Victor Sebastián-Pérez, Carlo De Giuli Morghen, Andrea Casiraghi, Paul M. Dunman, Ermanno Valoti and Valentina Straniero *

Summary

1-(4-biphenyl)-3-(2-methylpiperidin-1-yl)urea (1) 2
1-(3-biphenyl)-3-(2-methylpiperidin-1-yl)urea (2) 4
1-(2-biphenyl)-3-(2-methylpiperidin-1-yl)urea (3) 6
1-(3,5-dichlorophenyl)-3-(1,2,3,4-tetrahydroquinolin-1-yl)urea (4) 8
1-(3,5-dichlorophenyl)-3-(1,2,3,4-tetrahydroisoquinolin-1-yl)urea (5) 10
1-(4-*i*-propylphenoxyacetyl)-4-(3,5-dichlorobenzoyl)thiosemicarbazide (6) 12
1-(4-*i*-propylphenoxyacetyl)-4-(3-chlorobenzoyl)thiosemicarbazide (7) 14
1-(4-*i*-propylphenoxyacetyl)-4-(3-bromobenzoyl)thiosemicarbazide (8) 16
1-(4-*i*-propylphenoxyacetyl)-4-(3,5-dibromobenzoyl)thiosemicarbazide (9) 18
1-(4-biphenyloxyacetyl)-4-(3,5-dichlorobenzoyl)thiosemicarbazide (10) 20
3,5-dichloro-*N*-((2-(4-*i*-propylphenoxy)acetamido)methyl)benzamide (11) 22
3,5-dichloro-*N*-((2-(4-*i*-propylphenoxy)acetamido)ethyl)benzamide (12) 24
4-(3,5-dichlorobenzoylamino)-1-(4-*i*-propylphenoxyacetyl)piperidine (13) 26
4-(3,5-dichlorobenzoyl)-1-(4-*i*-propylphenoxyacetly)piperazine (14) 28

1-(4-biphenyl)-3-(2-methylpiperidin-1-yl)urea (1)

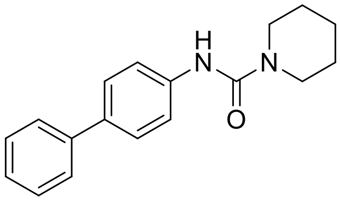

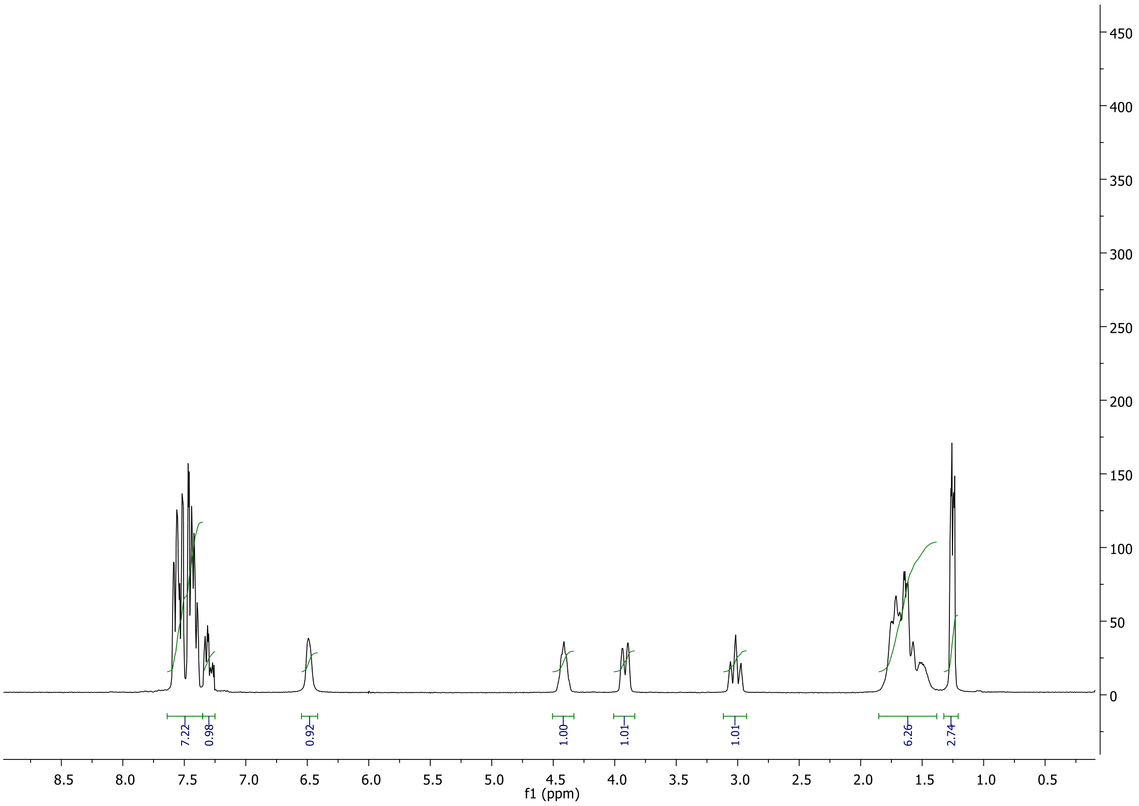

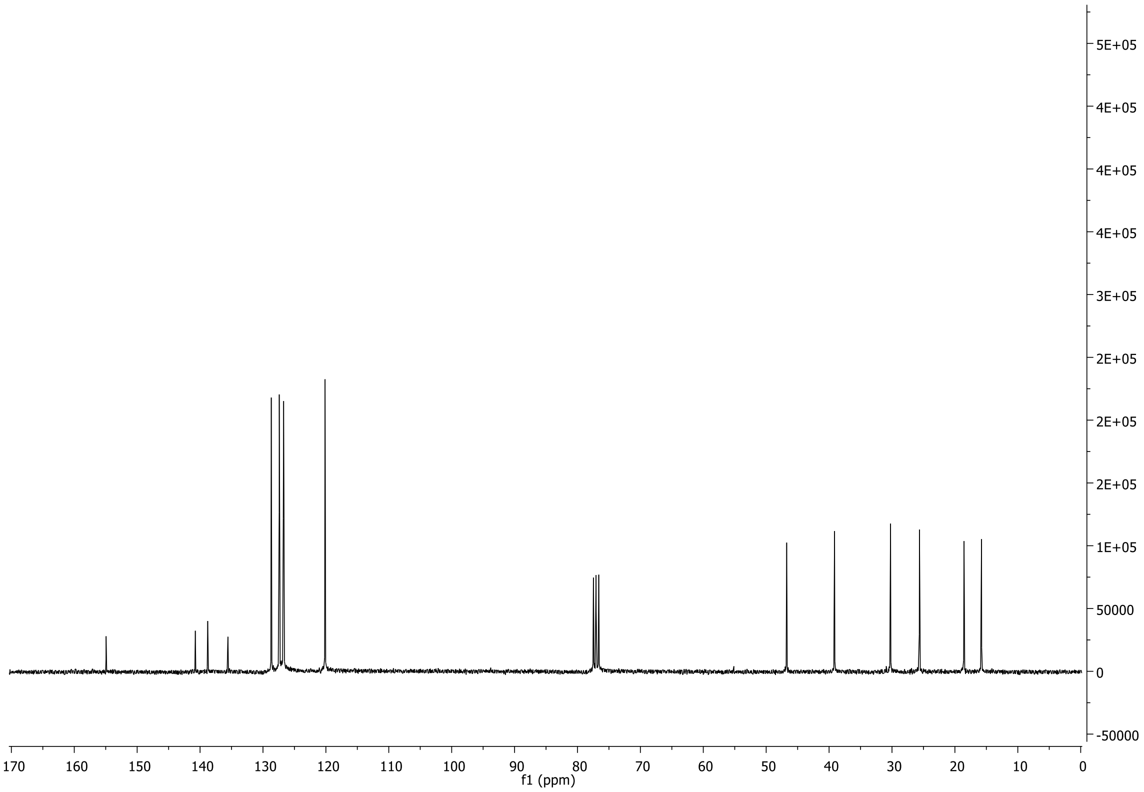

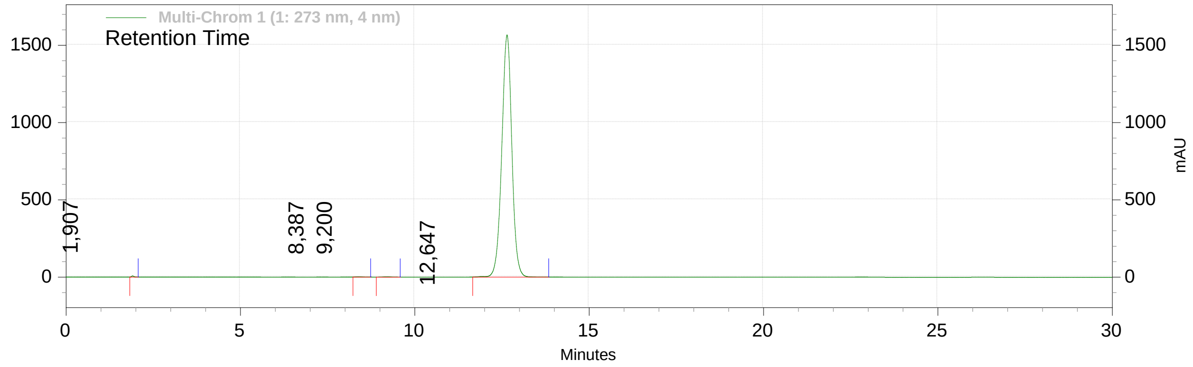

Multi-Chrom 1 (1: 273 nm, 4 nm)
Retention Time
1,907
8,387
9,200
12,647
1500
1000
500
0
mAU
0
5
10
15
20
25
30
Minutes

1-(3-biphenyl)-3-(2-methylpiperidin-1-yl)urea (2)

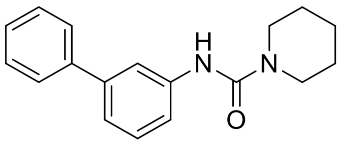

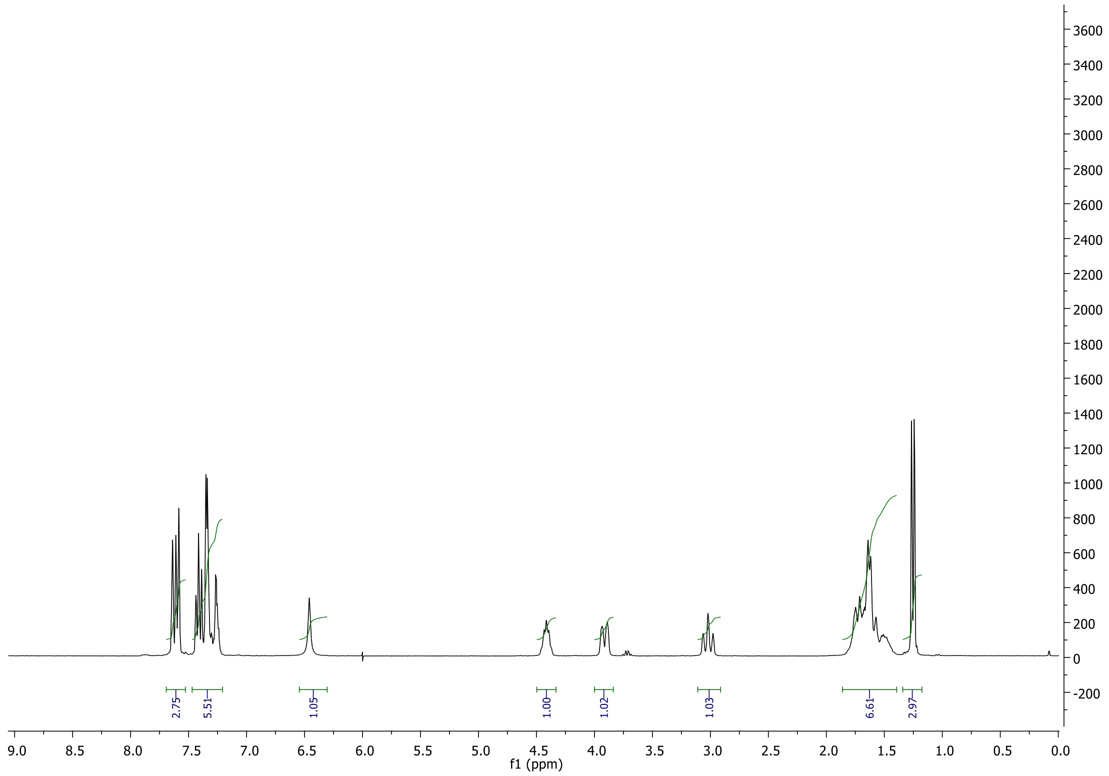

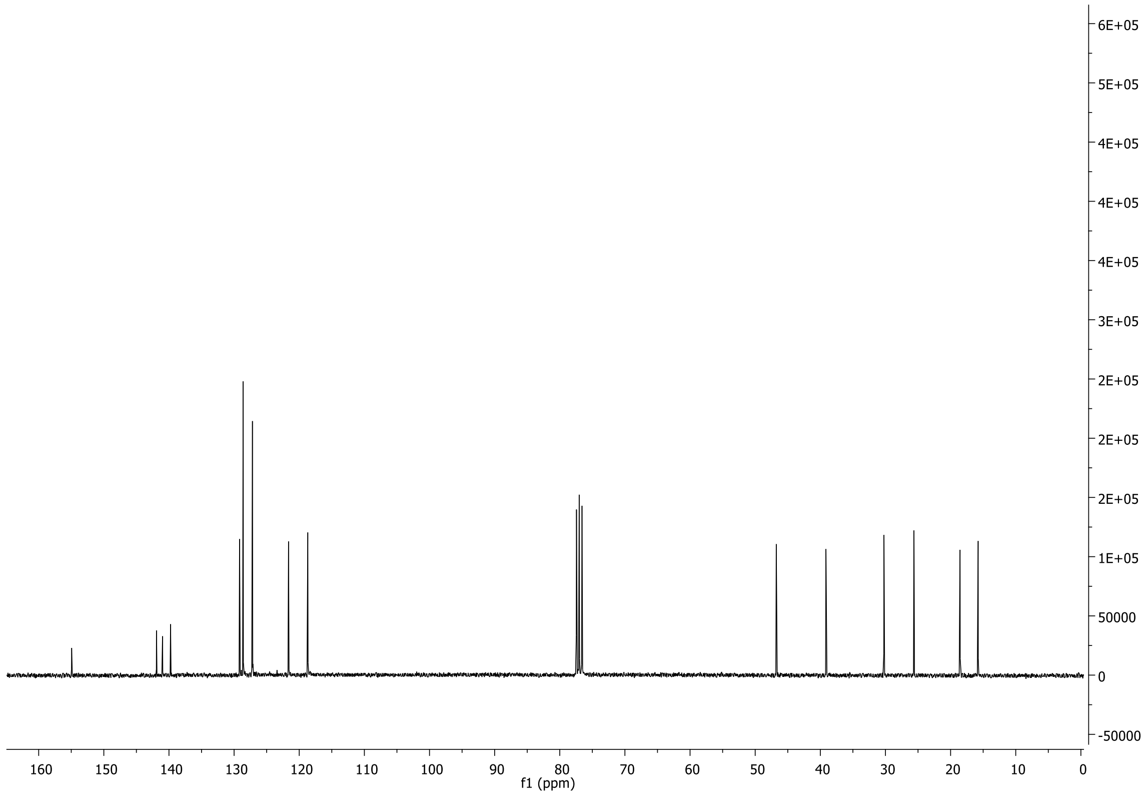

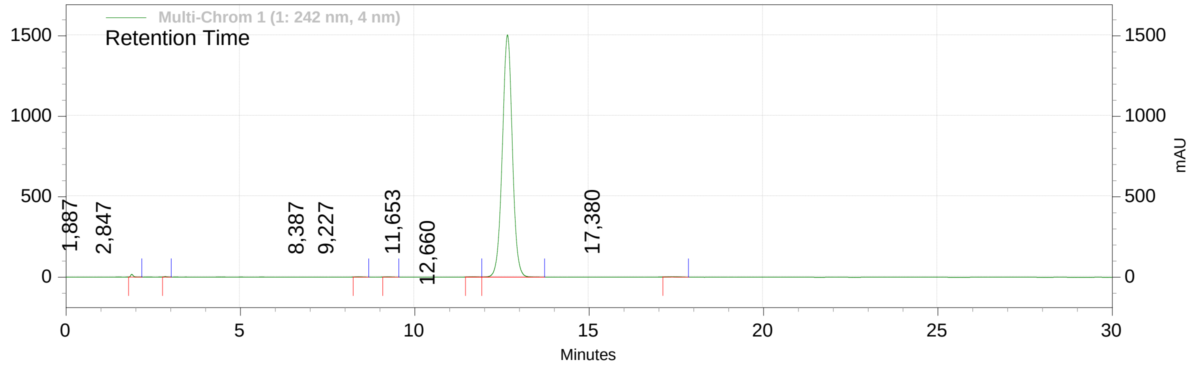
Multi-Chrom 1 (1: 242 nm, 4 nm)
Retention Time
1500
1000
500
0
1,887
2,847
8,387
9,227
11,653
12,660
17,380
0
5
10
15
20
25
30
Minutes
mAU

1-(2-biphenyl)-3-(2-methylpiperidin-1-yl)urea (3)

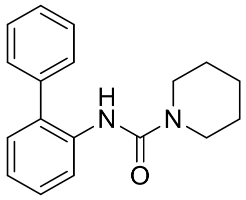

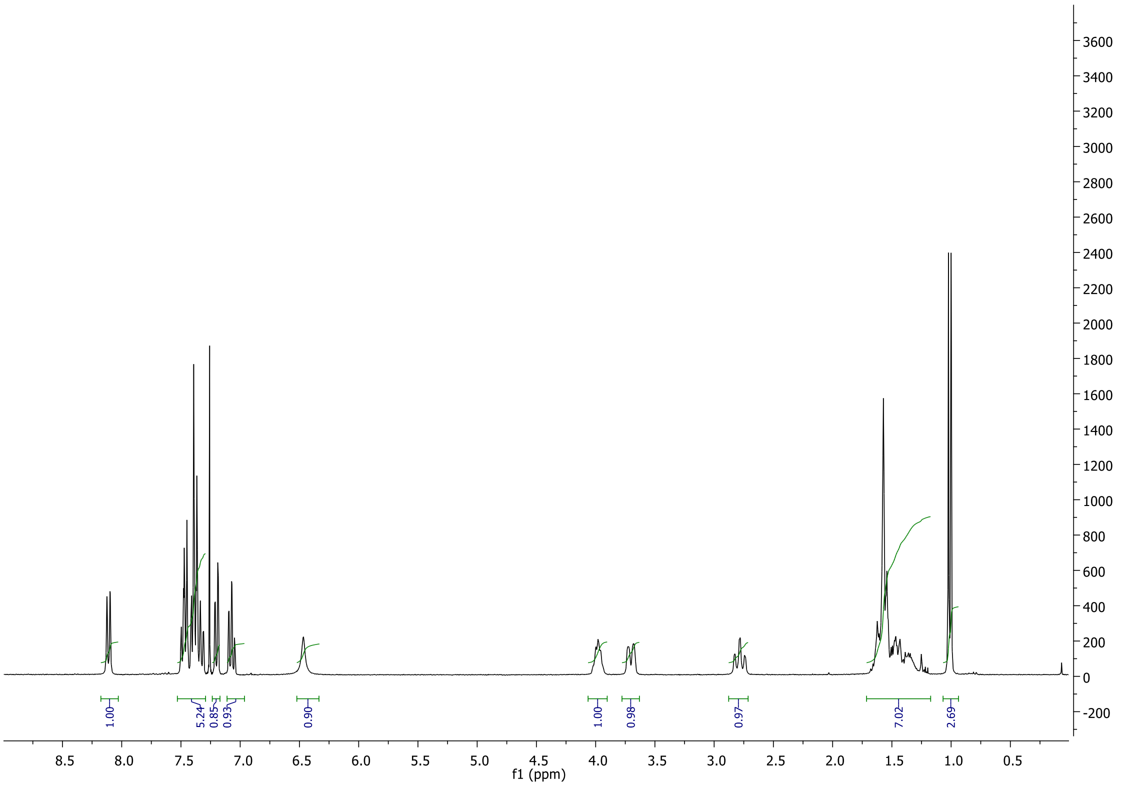

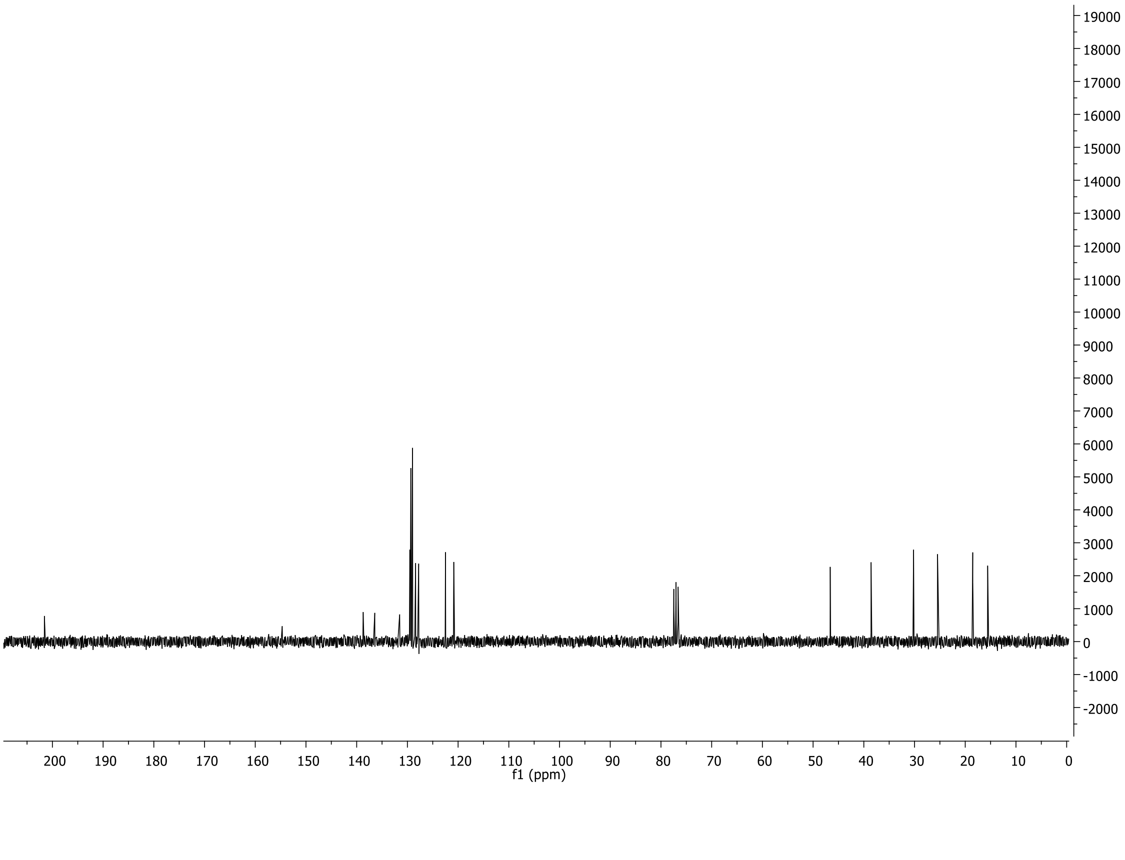

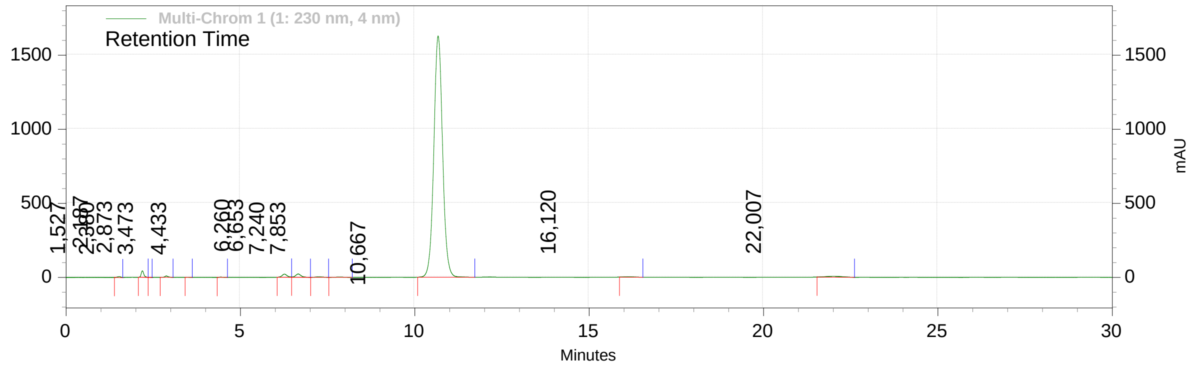

Multi-Chrom 1 (1: 230 nm, 4 nm)
Retention Time
1,527
2,187
2,380
2,873
3,473
4,433
6,260
6,653
7,240
7,853
10,667
16,120
22,007
mAU
Minutes

1-(3,5-dichlorophenyl)-3-(1,2,3,4-tetrahydroquinolin-1-yl)urea (4)

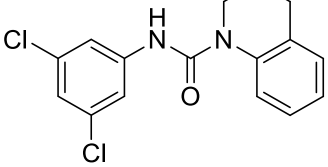

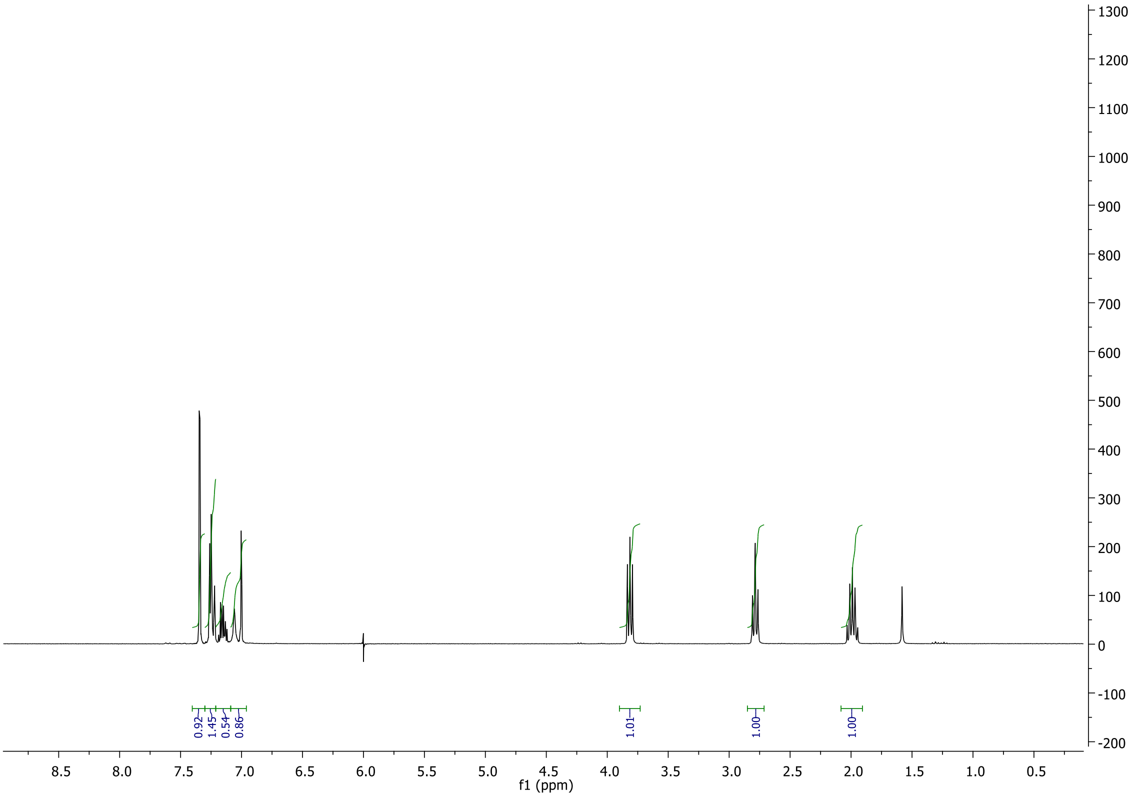

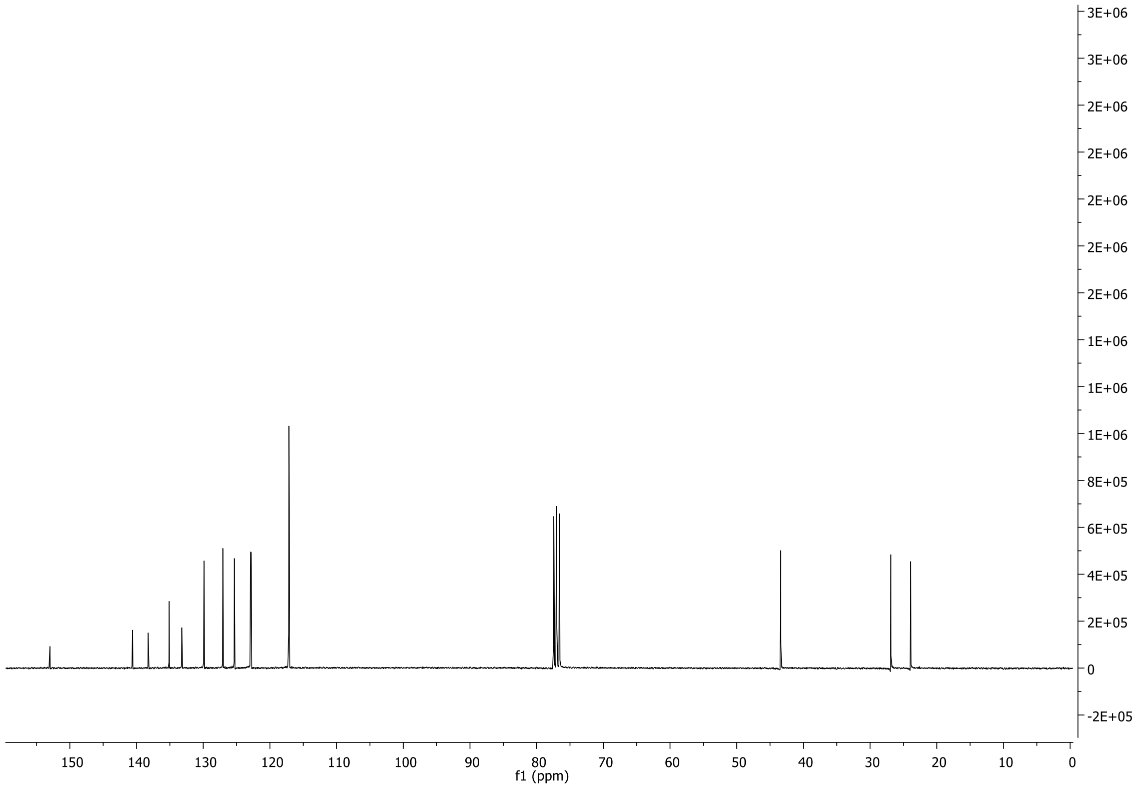

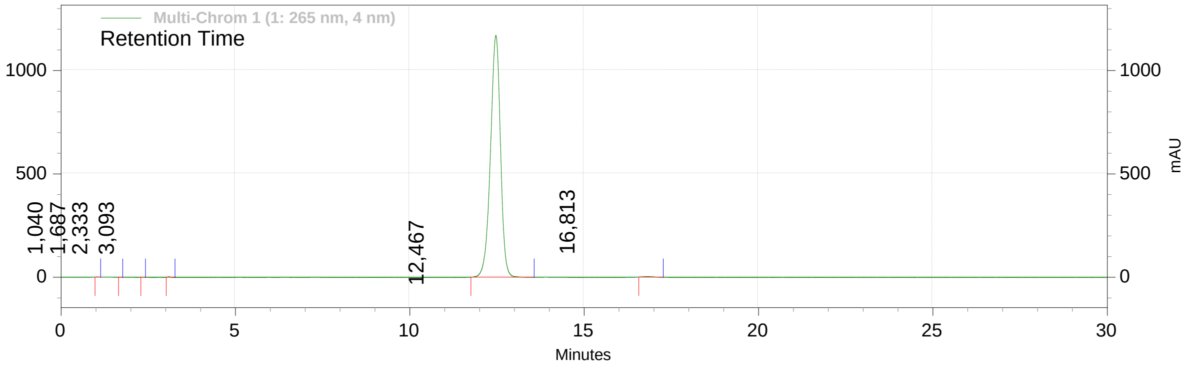

Multi-Chrom 1 (1: 265 nm, 4 nm)
Retention Time
1000
500
0
mAU
1,040
1,687
2,333
3,093
12,467
16,813
0
5
10
15
20
25
30
Minutes

1-(3,5-dichlorophenyl)-3-(1,2,3,4-tetrahydroisoquinolin-1-yl)urea (5)

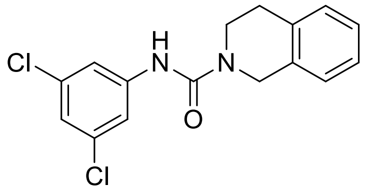

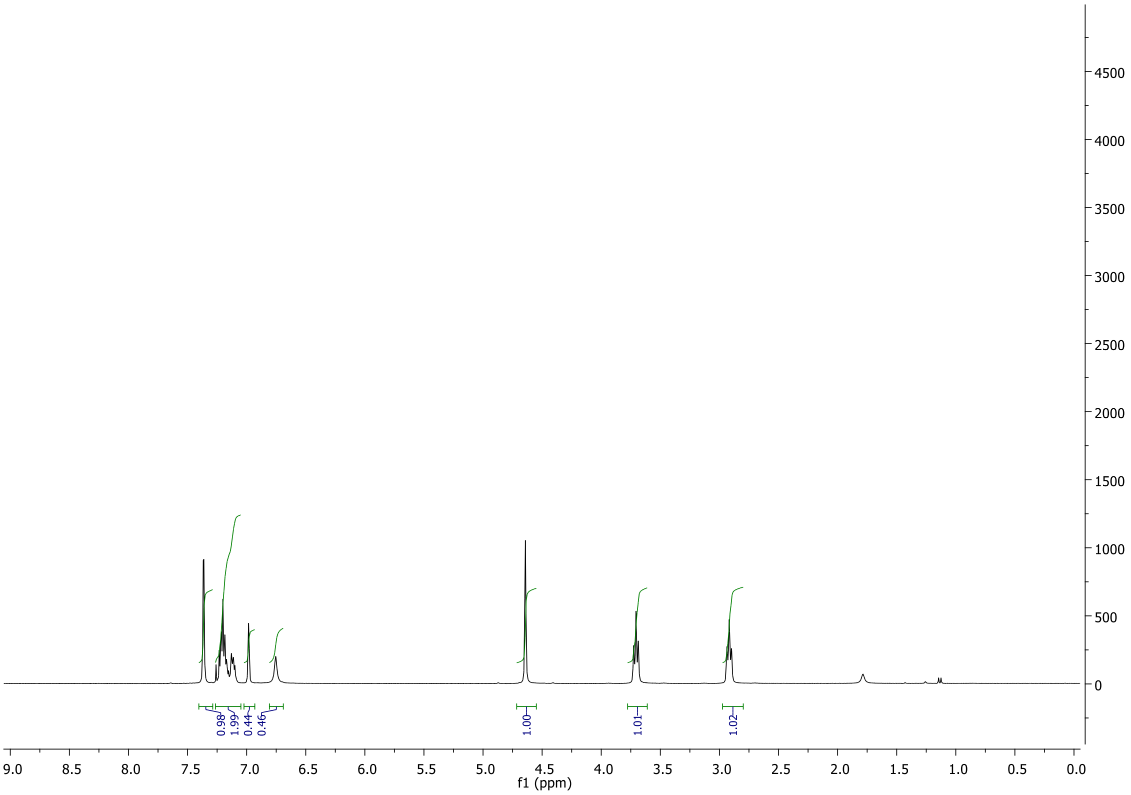

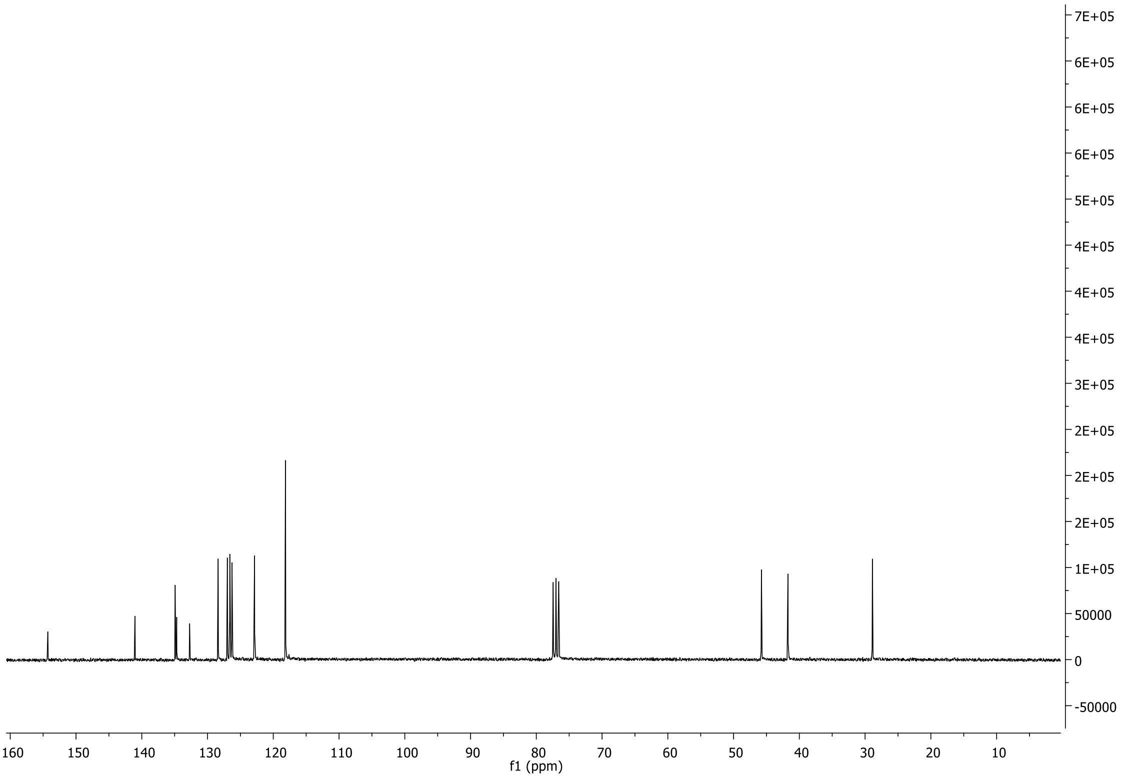

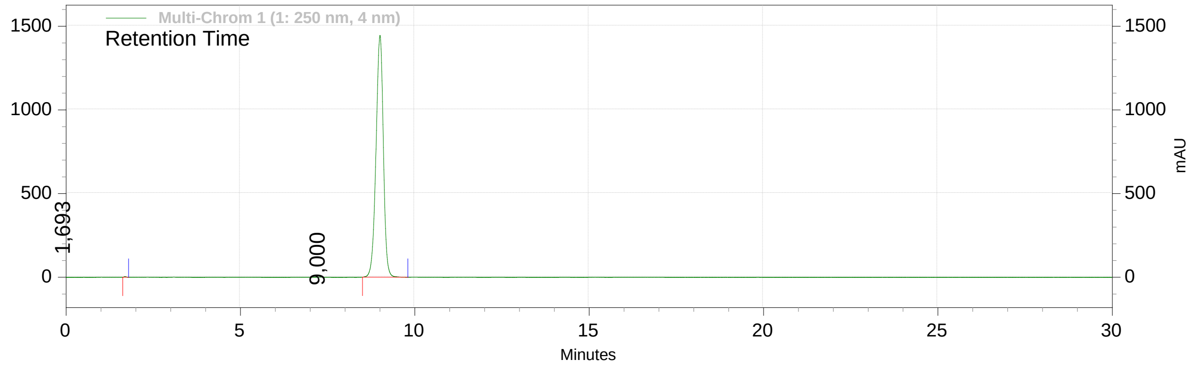

Multi-Chrom 1 (1: 250 nm, 4 nm)
Retention Time
1,693
9,000
1500
1000
500
0
mAU
0
5
10
15
20
25
30
Minutes

1-(4-*i*-propylphenoxyacetyl)-4-(3,5-dichlorobenzoyl)thiosemicarbazide (6)

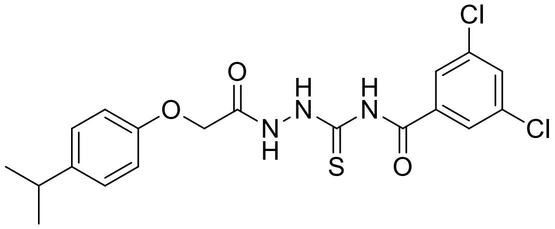

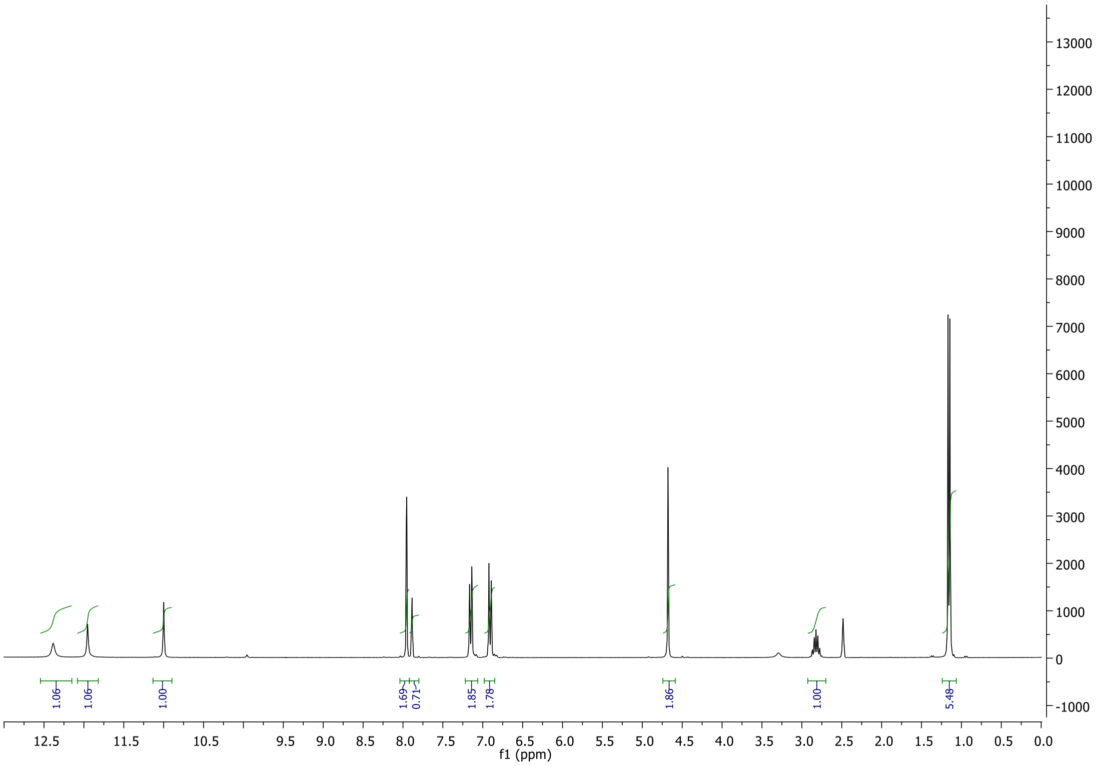

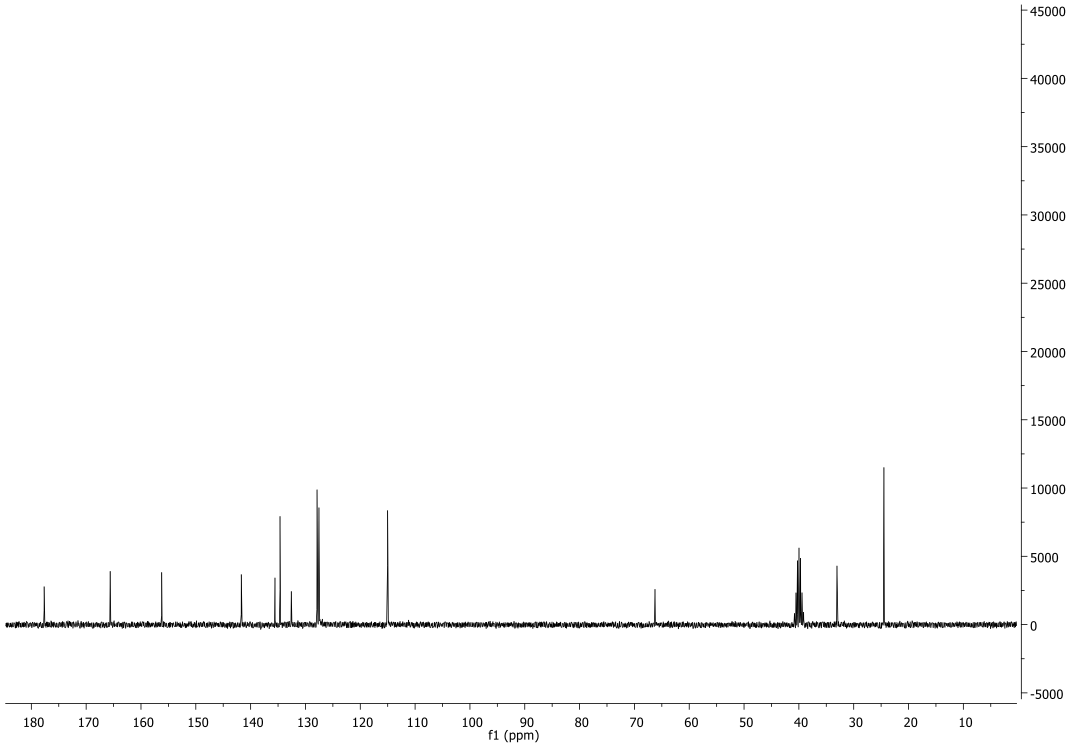

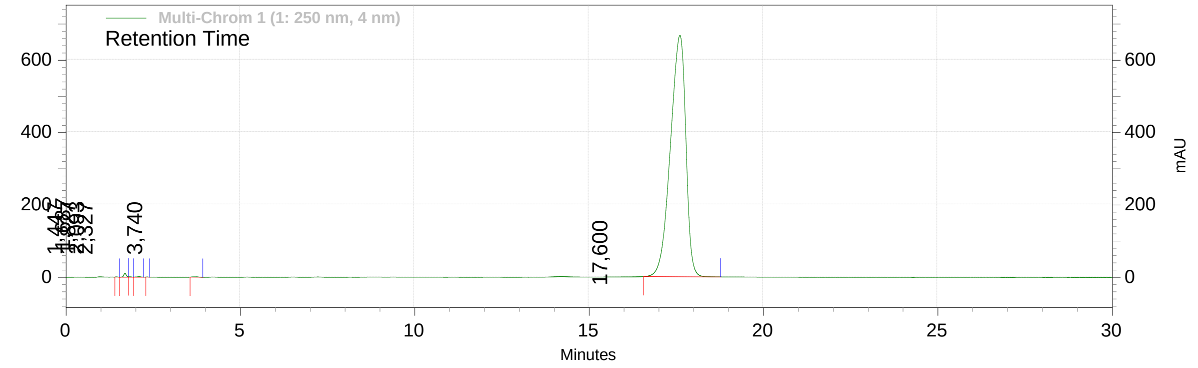

Multi-Chrom 1 (1: 250 nm, 4 nm)
Retention Time
600
400
200
0
mAU
3,740
17,600
2,327
0
5
10
15
20
25
30
Minutes

1-(4-*i*-propylphenoxyacetyl)-4-(3-chlorobenzoyl)thiosemicarbazide (7)

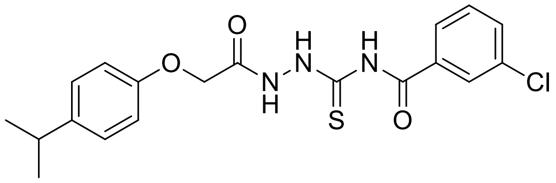

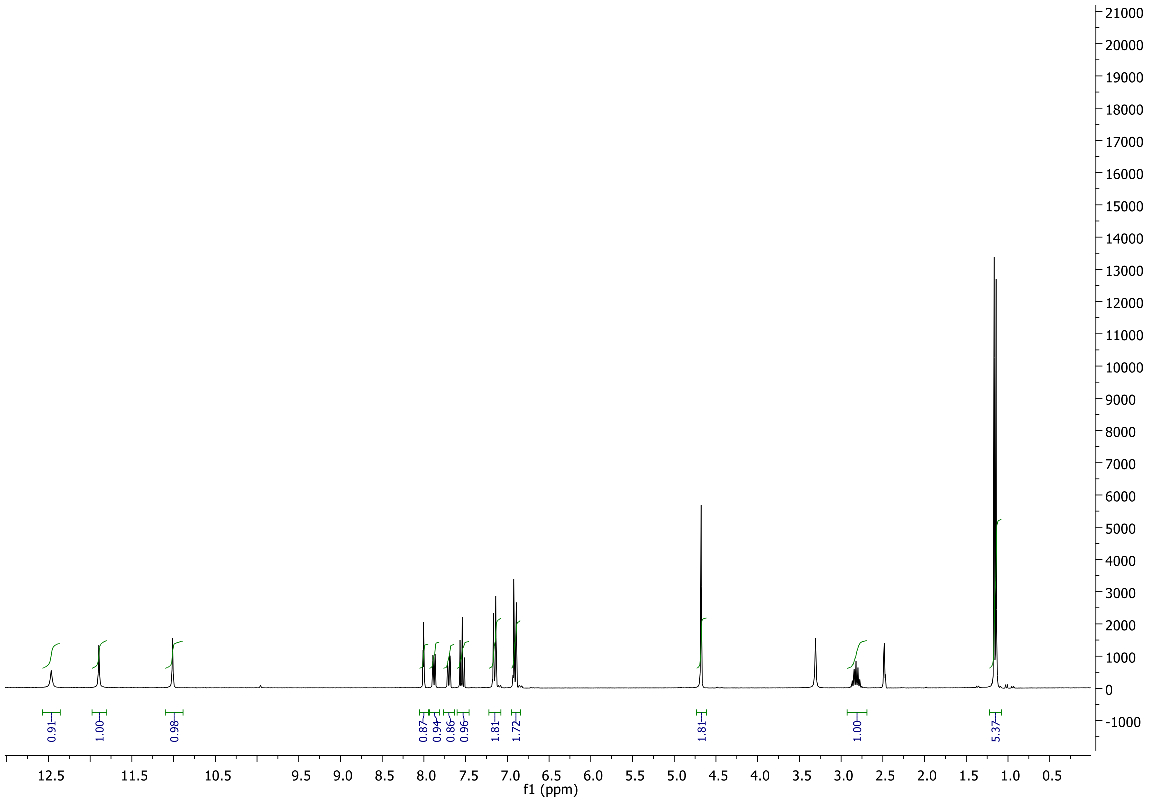

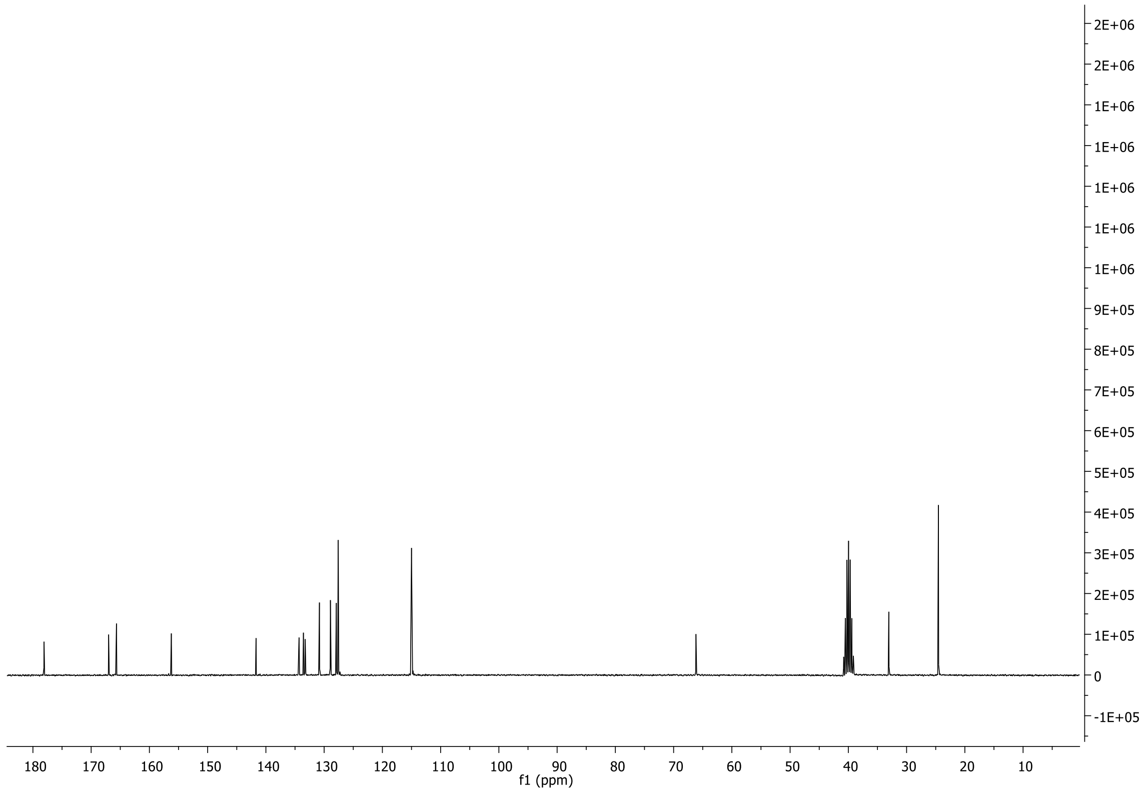

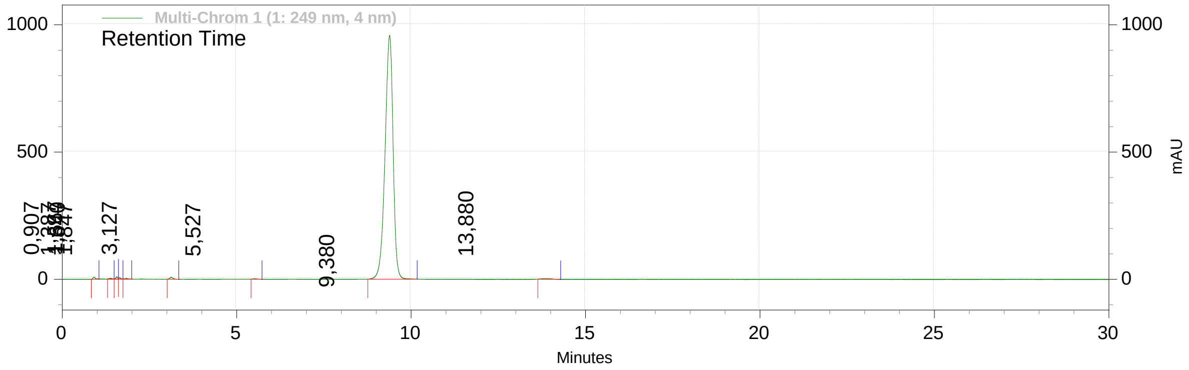

Multi-Chrom 1 (1: 249 nm, 4 nm)
Retention Time
1000
500
0
mAU
0,907
3,127
5,527
9,380
13,880
0
5
10
15
20
25
30
Minutes

1-(4-*i*-propylphenoxyacetyl)-4-(3-bromobenzoyl)thiosemicarbazide (8)

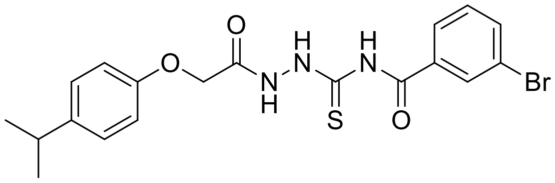

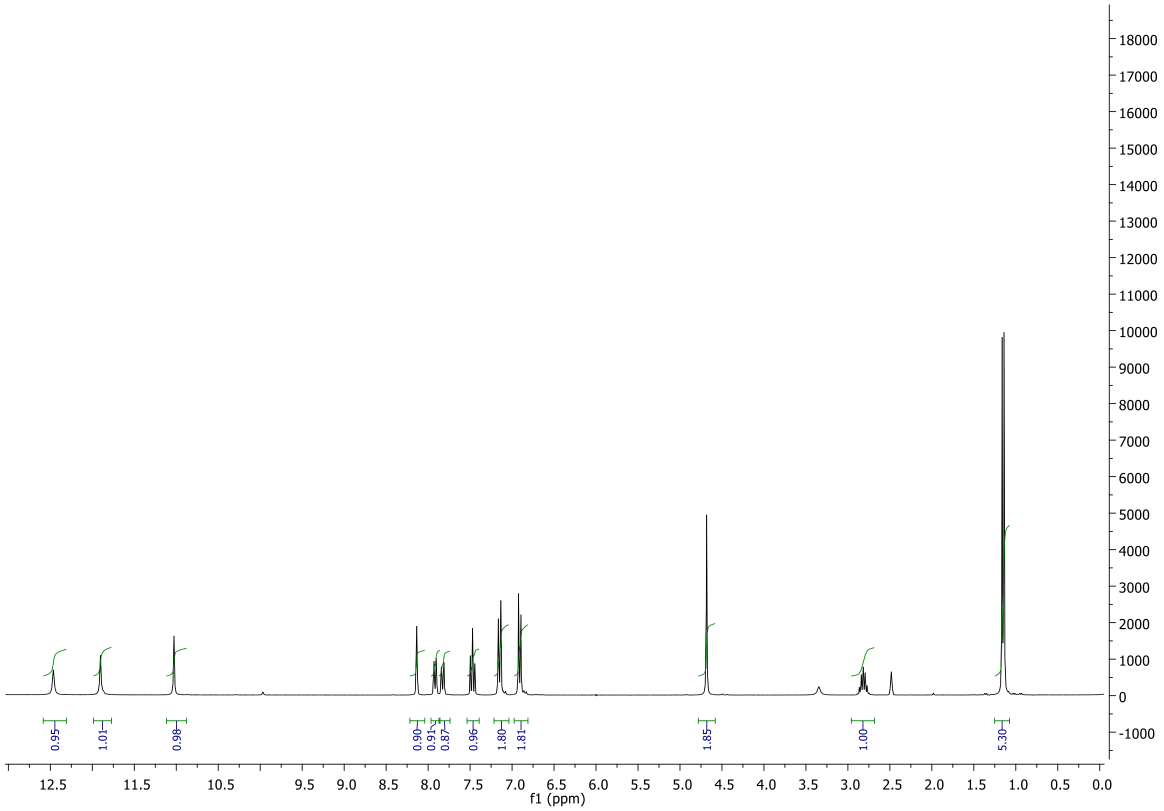

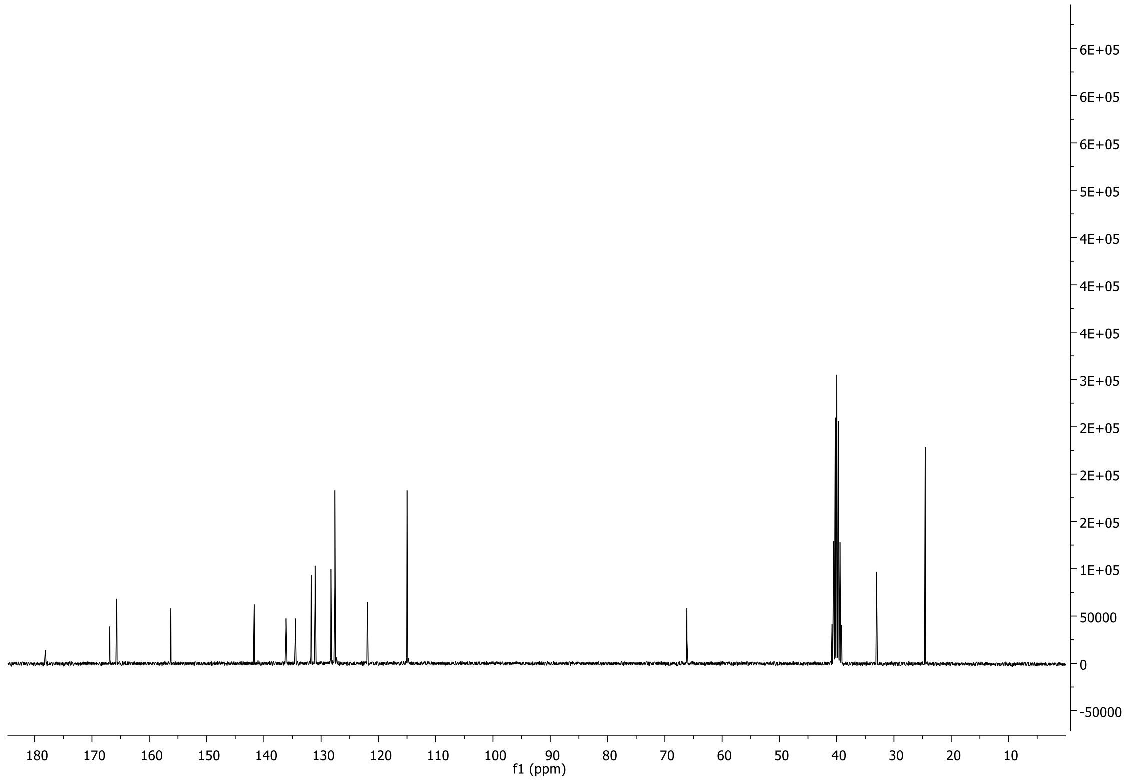

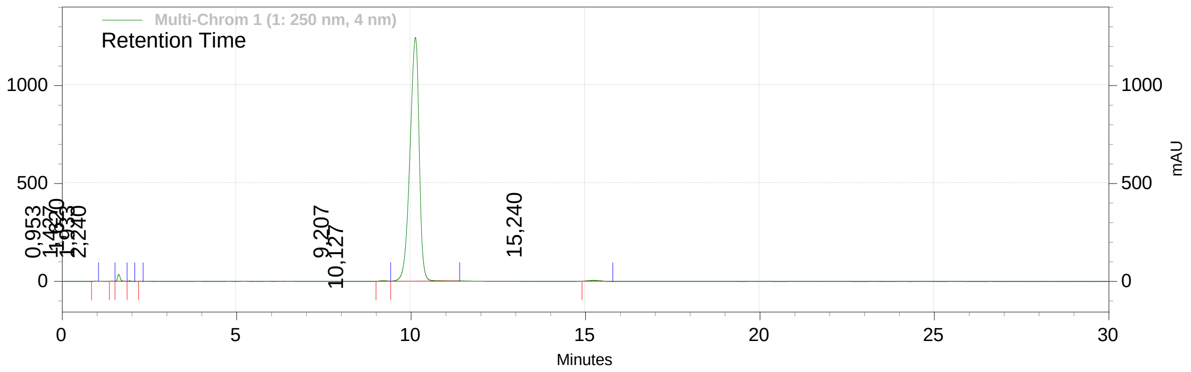

Multi-Chrom 1 (1: 250 nm, 4 nm)
Retention Time
0,953
1,437
1,620
1,933
2,240
9,207
10,127
15,240
mAU
Minutes

1-(4-*i*-propylphenoxyacetyl)-4-(3,5-dibromobenzoyl)thiosemicarbazide (9)

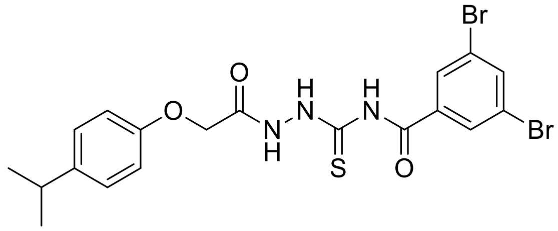

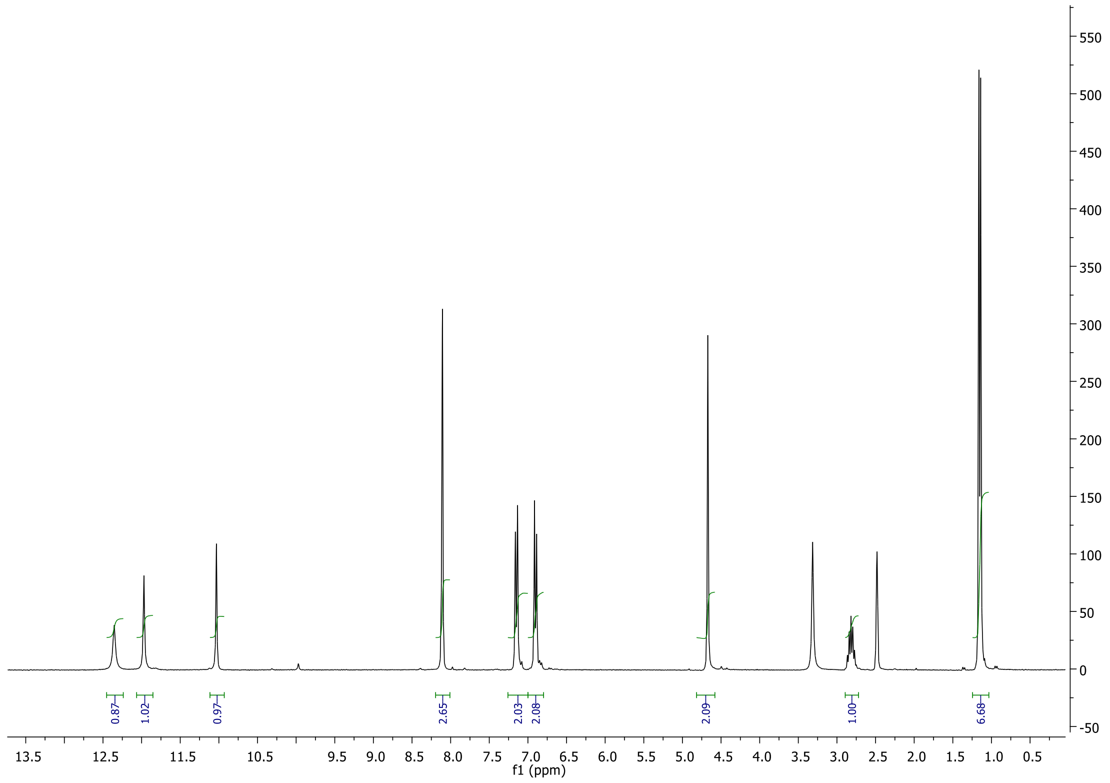

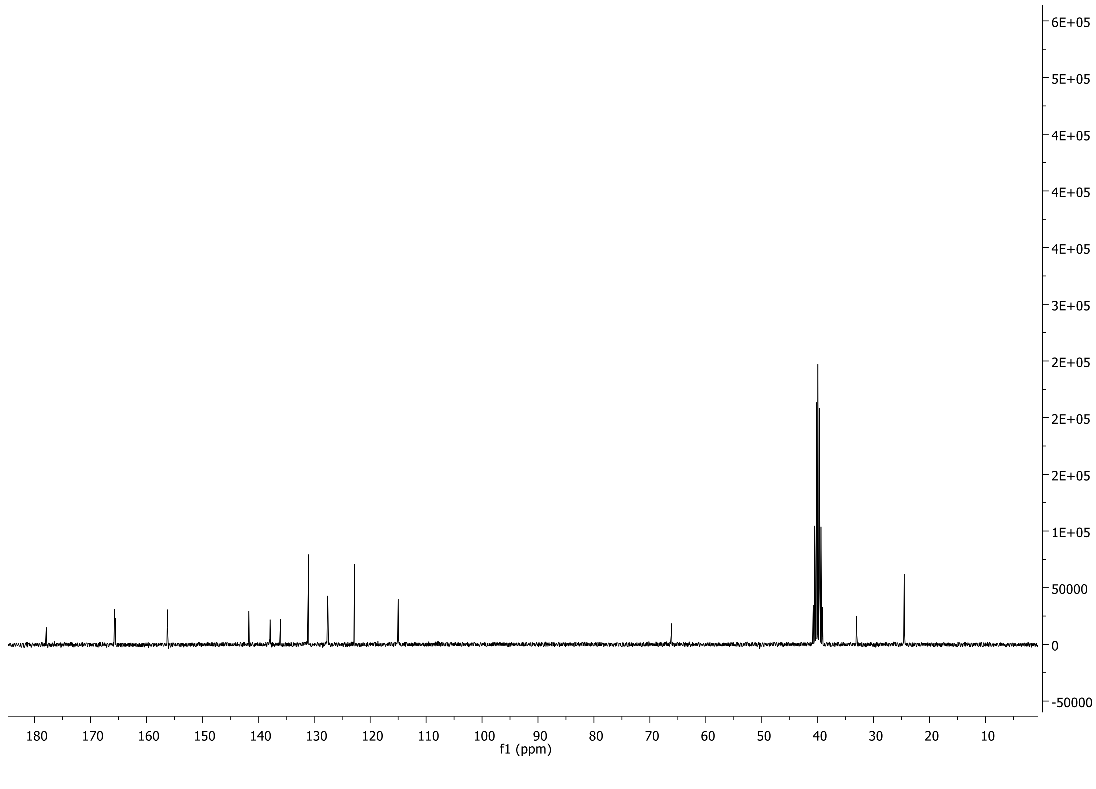

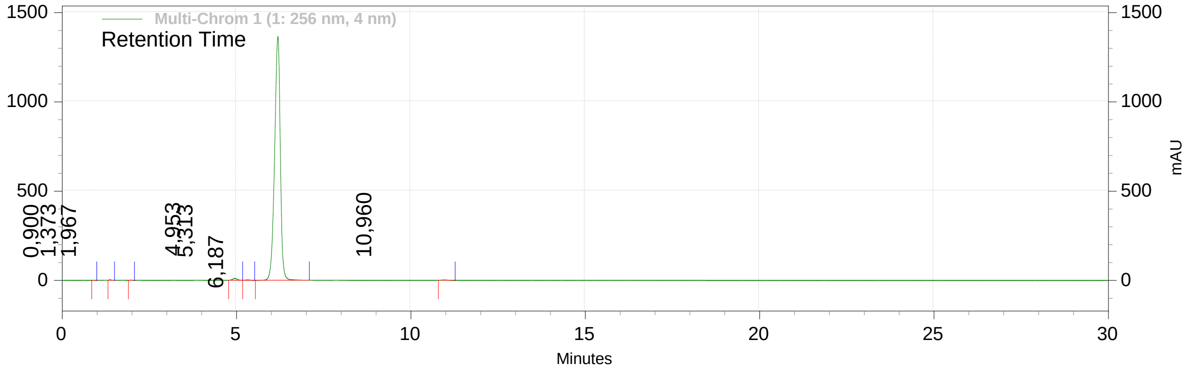

Multi-Chrom 1 (1: 256 nm, 4 nm)
Retention Time
0,900
1,373
1,967
4,953
5,313
6,187
10,960
mAU
Minutes

1-(4-biphenyloxyacetyl)-4-(3,5-dichlorobenzoyl)thiosemicarbazide (10)

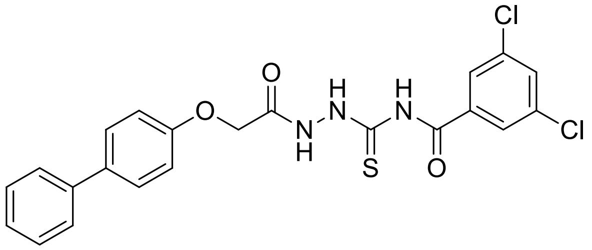

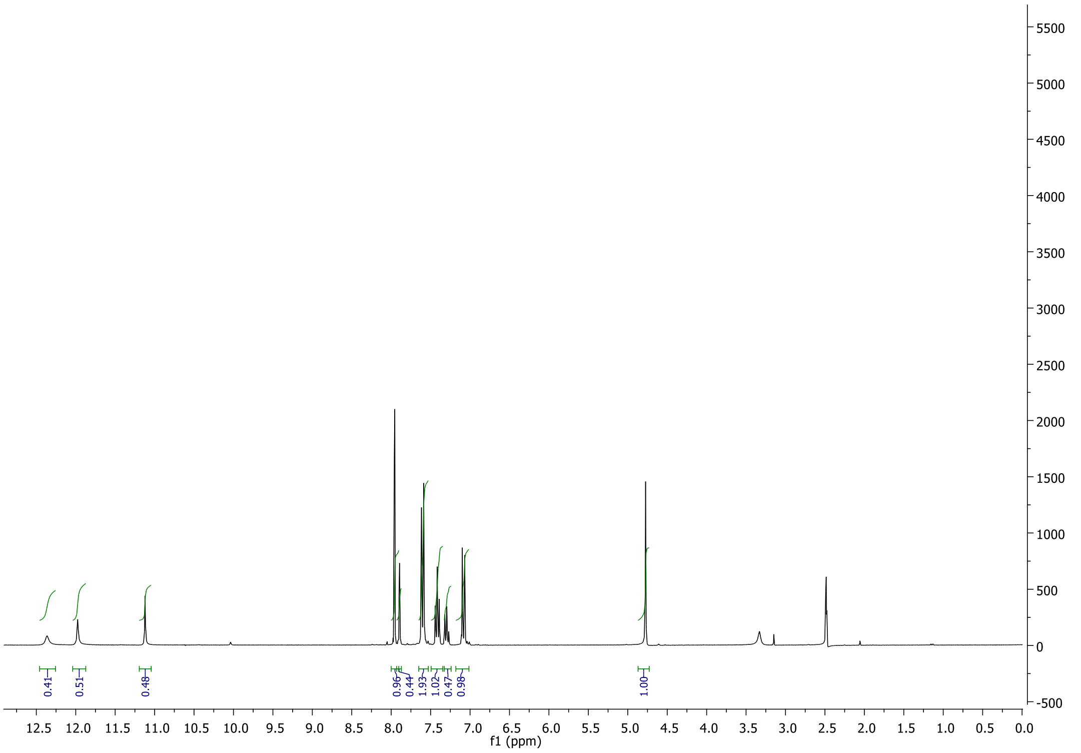

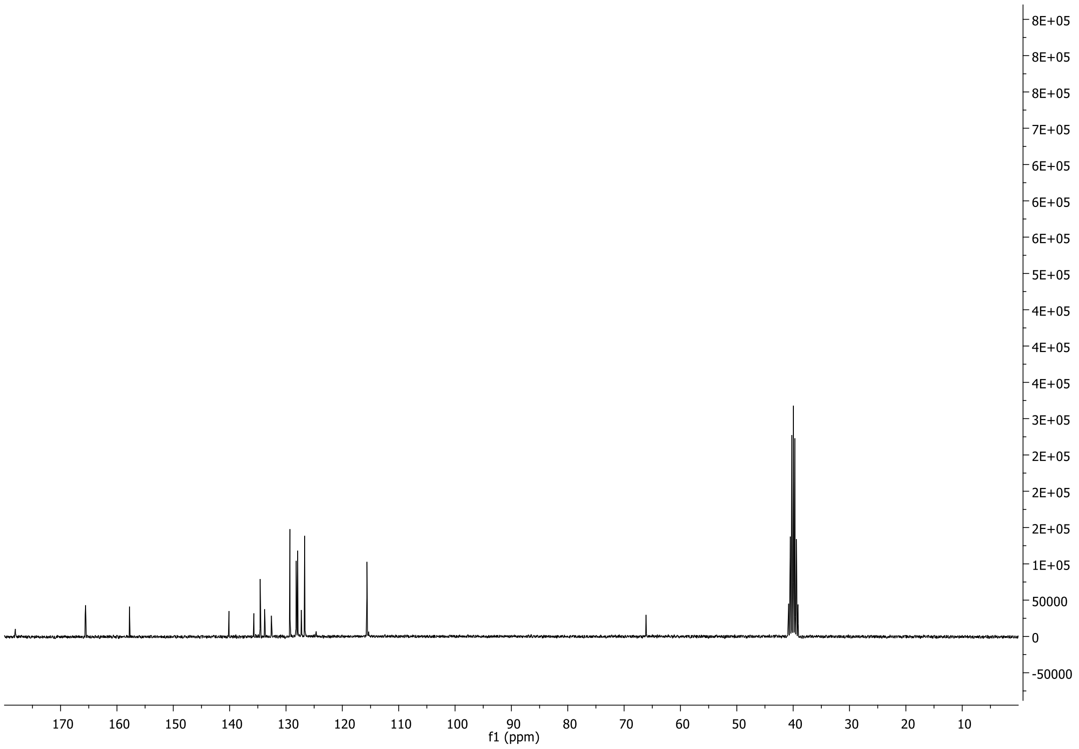

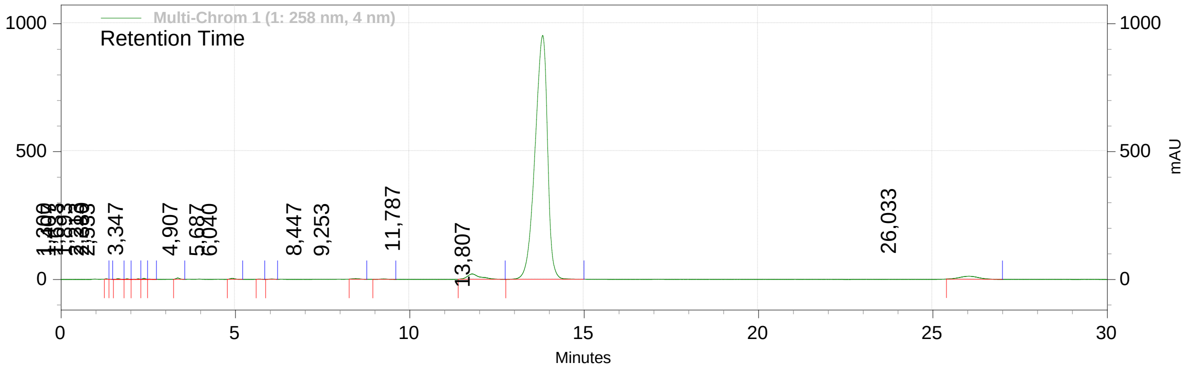

Multi-Chrom 1 (1: 258 nm, 4 nm)
Retention Time
1000
500
0
mAU
3,347
4,907
5,687
6,040
8,447
9,253
11,787
13,807
26,033
0
5
10
15
20
25
30
Minutes

3,5-dichloro-*N*-((2-(4-*i*-propylphenoxy)acetamido)methyl)benzamide (11)

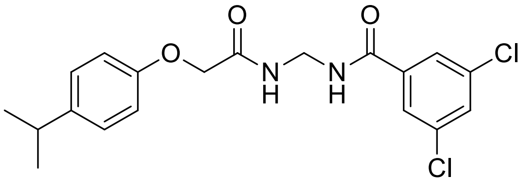

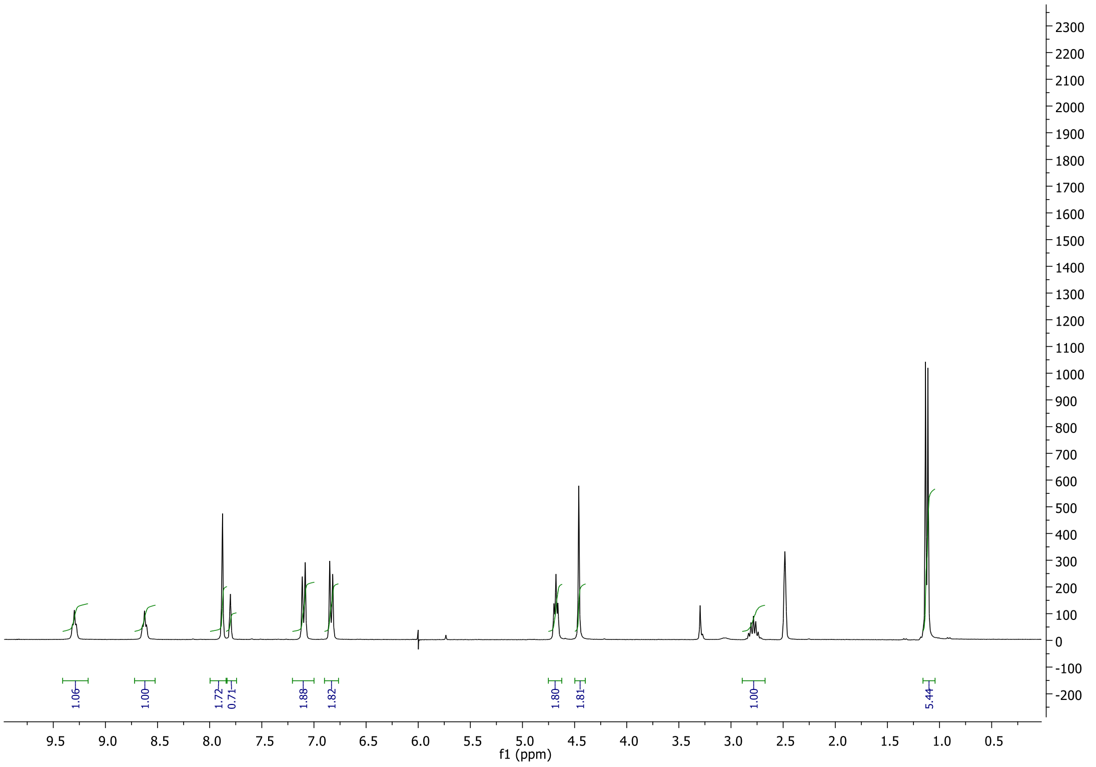

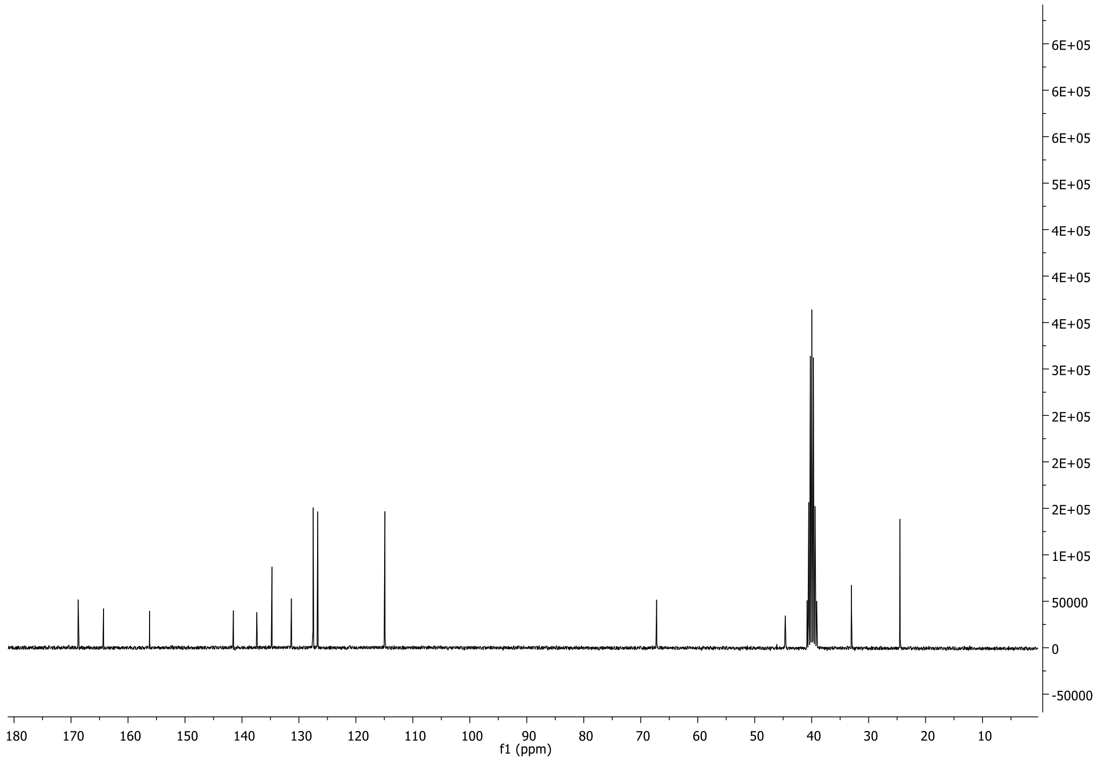

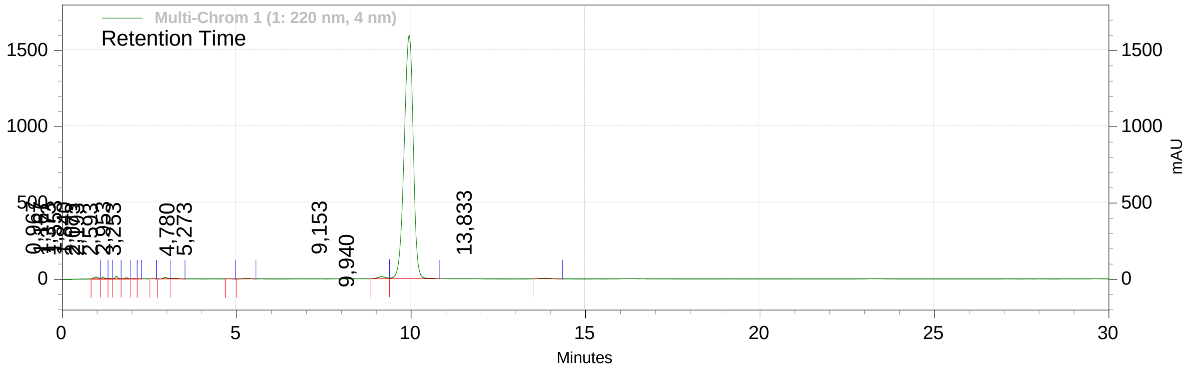
Multi-Chrom 1 (1: 220 nm, 4 nm)
Retention Time
4,780
5,273
9,153
9,940
13,833
mAU
Minutes

3,5-dichloro-*N*-((2-(4-*i*-propylphenoxy)acetamido)ethyl)benzamide (12)

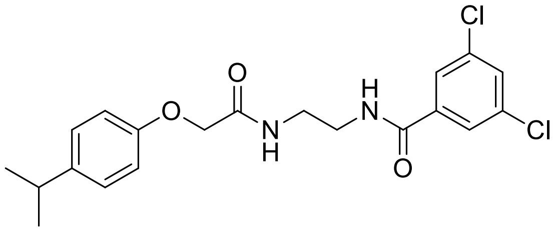

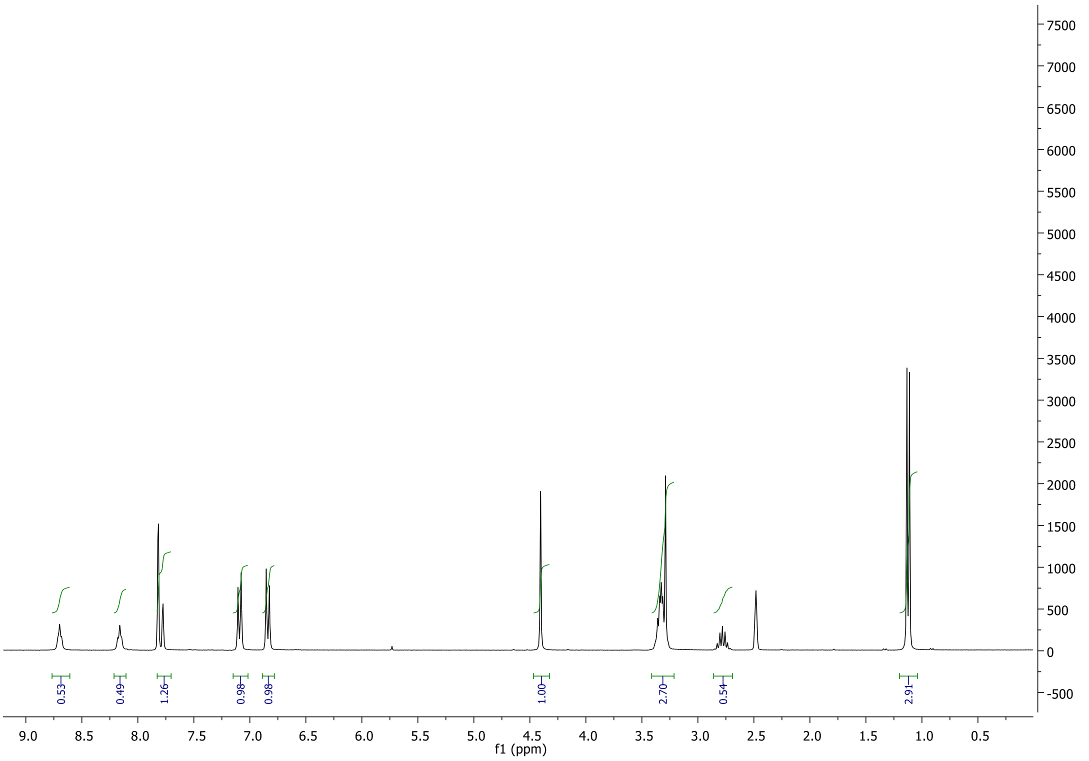

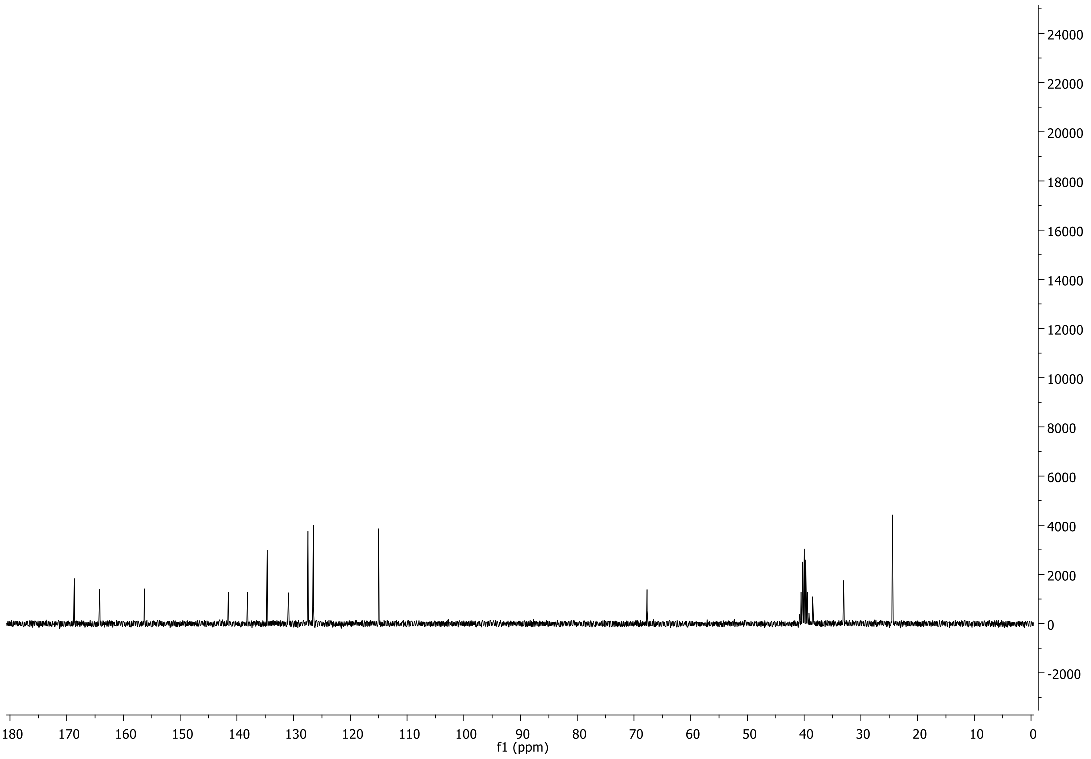

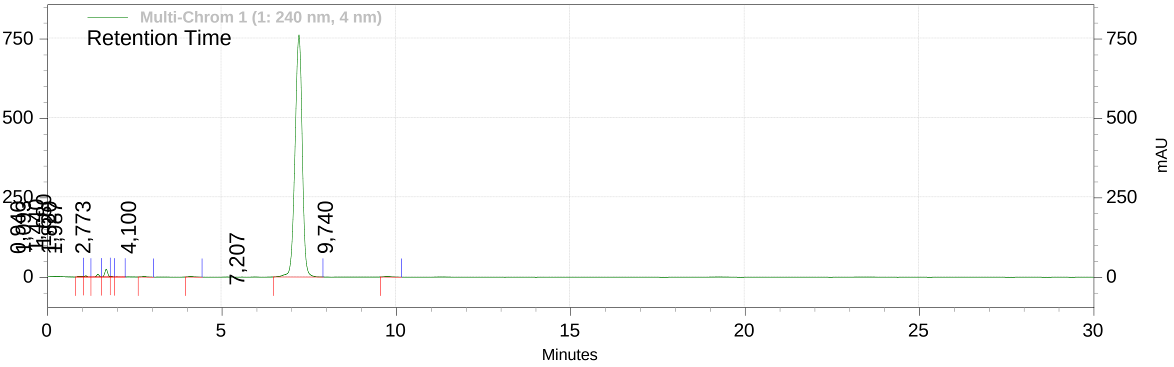

Multi-Chrom 1 (1: 240 nm, 4 nm)
Retention Time
2,773
4,100
7,207
9,740
mAU
Minutes

4-(3,5-dichlorobenzoylamino)-1-(4-*i*-propylphenoxyacetyl)piperidine (13)

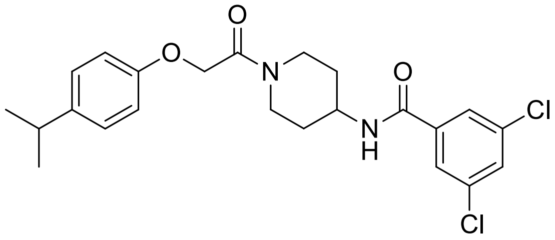

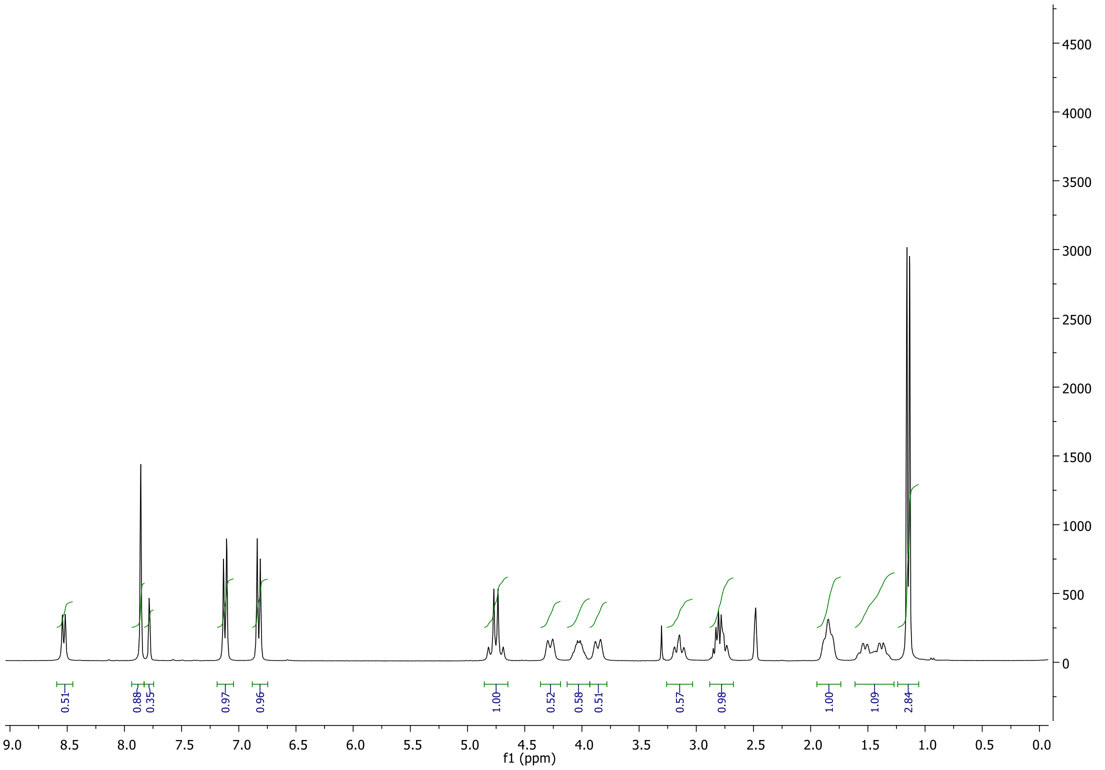

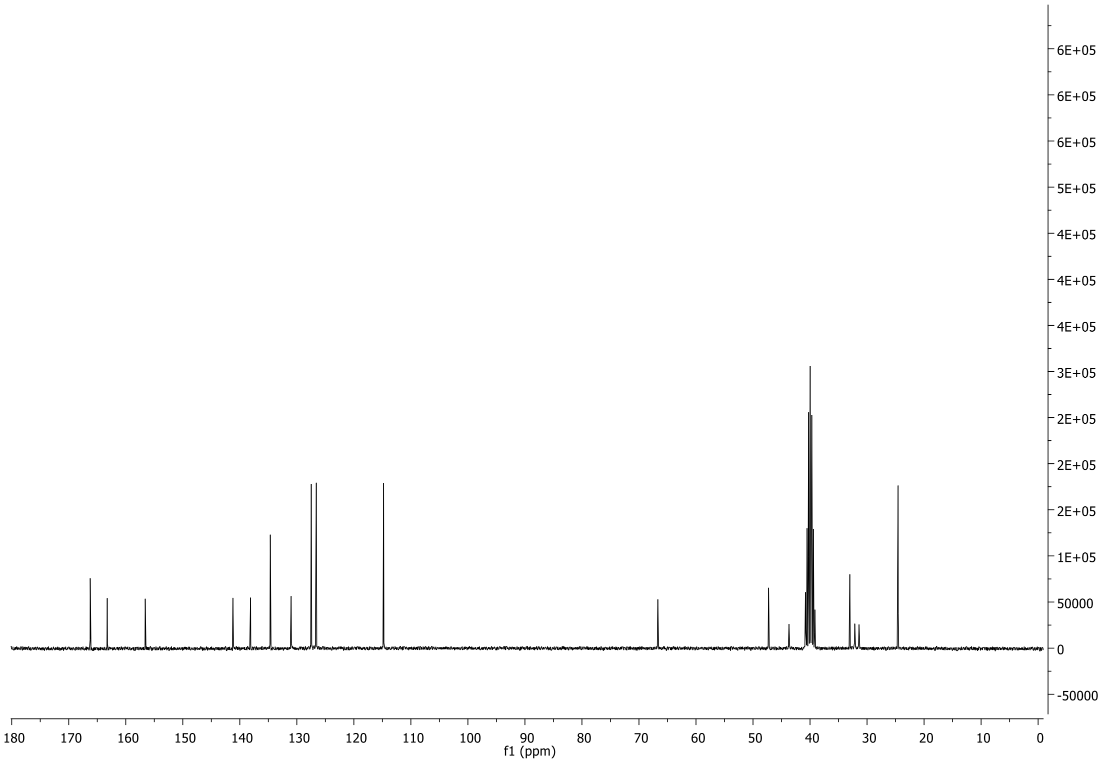

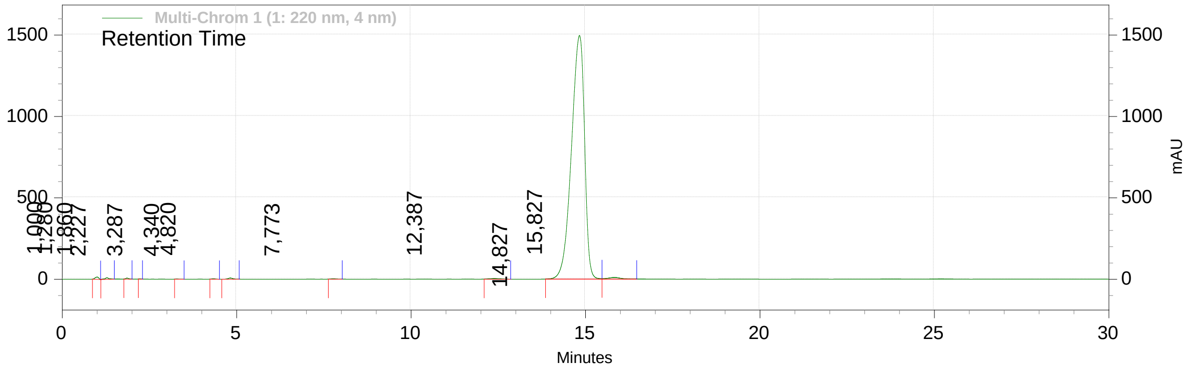

Multi-Chrom 1 (1: 220 nm, 4 nm)
Retention Time
1500
1000
500
0
mAU
1,000
1,280
1,860
2,227
3,287
4,340
4,820
7,773
12,387
14,827
15,827
0
5
10
15
20
25
30
Minutes

4-(3,5-dichlorobenzoyl)-1-(4-*i*-propylphenoxyacetly)piperazine (14)

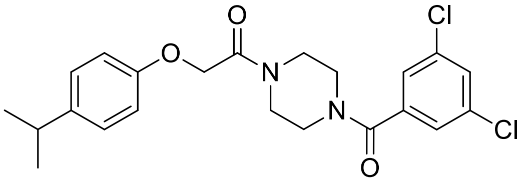

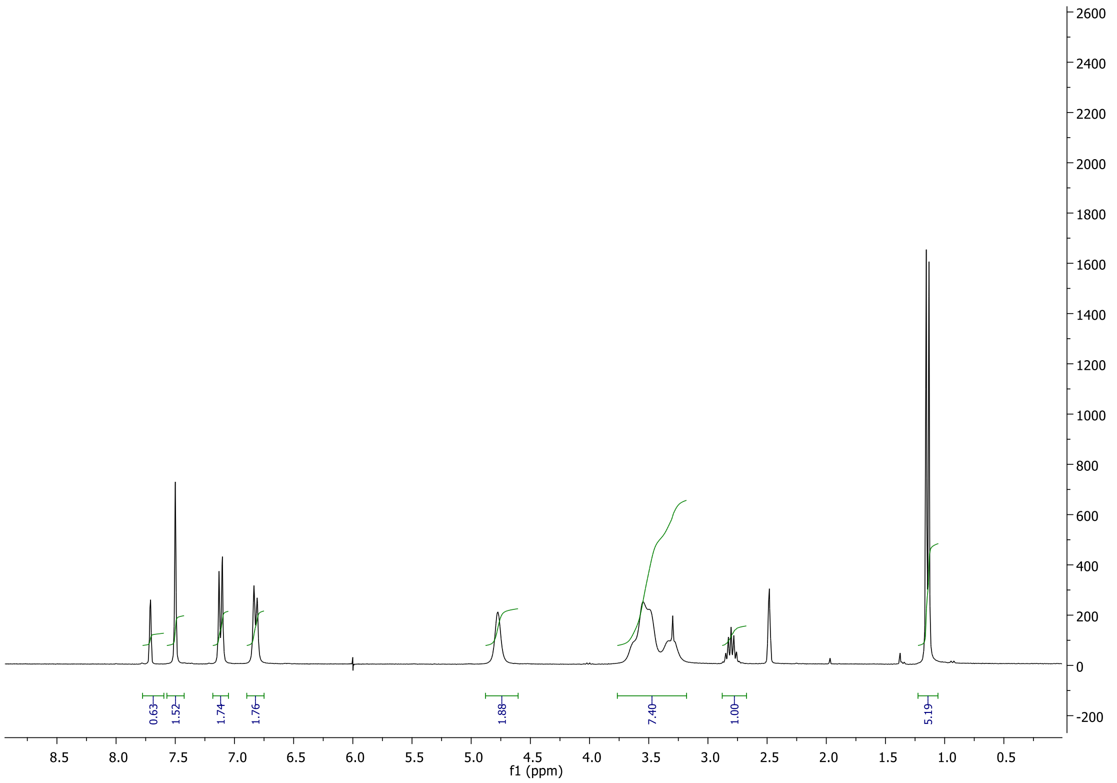

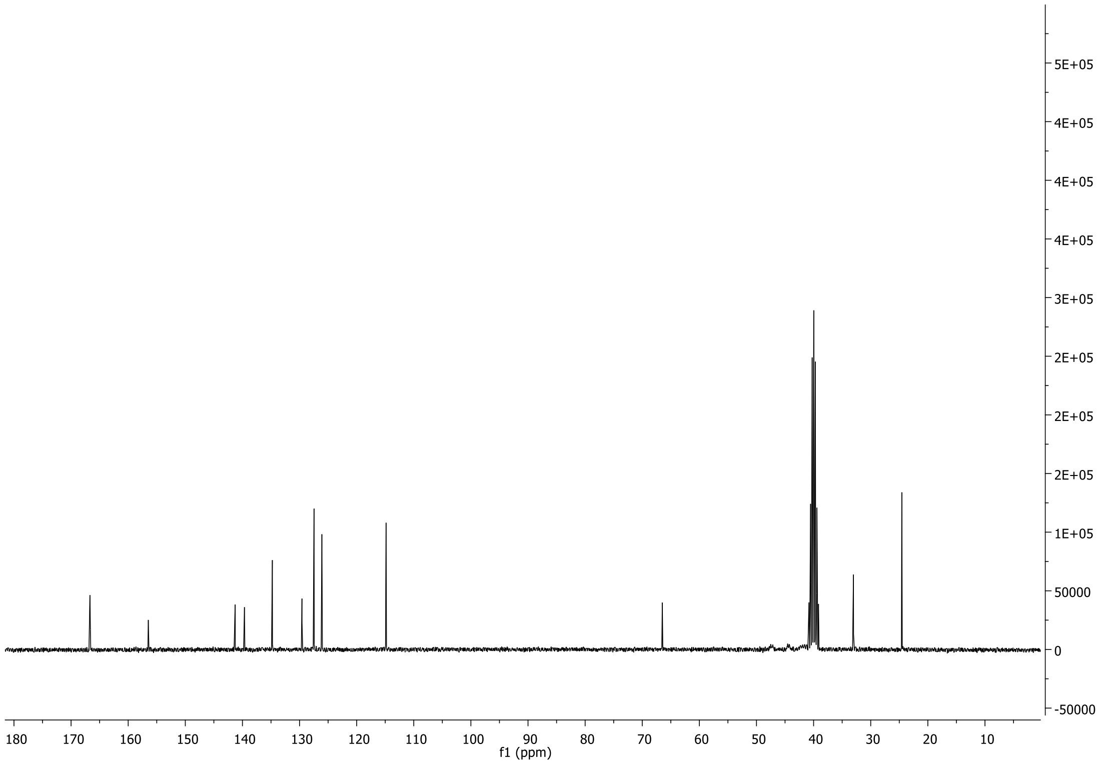

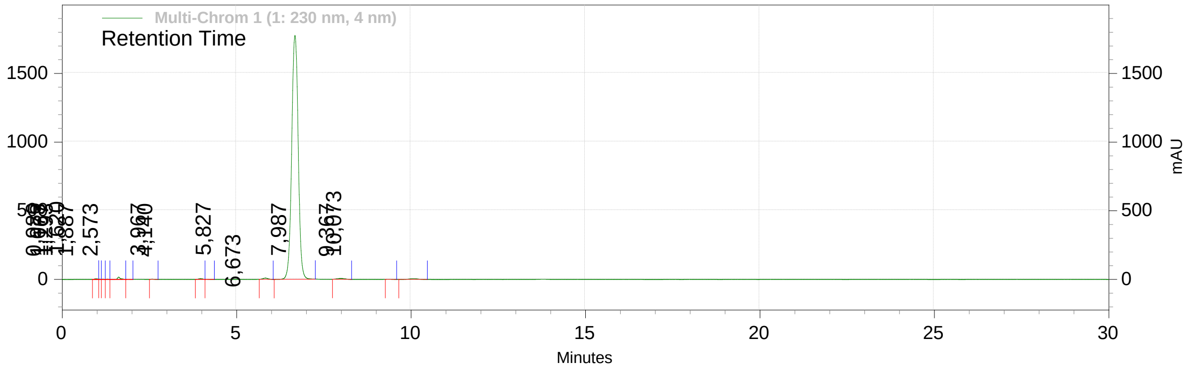

Multi-Chrom 1 (1: 230 nm, 4 nm)
Retention Time
1500
1000
500
0
2,573
3,967
4,140
5,827
6,673
7,987
9,367
10,073
0
5
10
15
20
25
30
Minutes
mAU
